# Matrix Degradation in Human Immunodeficiency Virus Type 1–Associated Tuberculosis and Tuberculosis Immune Reconstitution Inflammatory Syndrome: A Prospective Observational Study

**DOI:** 10.1093/cid/cix231

**Published:** 2017-05-05

**Authors:** Naomi F Walker, Katalin A Wilkinson, Graeme Meintjes, Liku B Tezera, Rene Goliath, Janique M Peyper, Rebecca Tadokera, Charles Opondo, Anna K Coussens, Robert J Wilkinson, Jon S Friedland, Paul T Elkington

**Affiliations:** 1 Wellcome Centre for Infectious Diseases Research in Africa, Institute of Infectious Disease and Molecular Medicine, University of Cape Town, Observatory, South Africa;; 2 Infectious Diseases and Immunity, and Imperial College Wellcome Trust Centre for Global Health, Imperial College London, United Kingdom;; 3 Department of Medicine, University of Cape Town, Observatory, South Africa;; 4 Department of Clinical Research, London School of Hygiene and Tropical Medicine,; 5 Francis Crick Institute, London, and; 6 National Institute for Health Research Respiratory Biomedical Research Unit, Clinical and Experimental Sciences Academic Unit, Faculty of Medicine, University of Southampton, United Kingdom;; 7 Applied Proteomics and Chemical Biology Group, Department of Integrative Biomedical Sciences, and; 8 Institute of Infectious Disease and Molecular Medicine, University of Cape Town, Observatory, and; 9 HIV/AIDS, Sexually Transmitted Infections and Tuberculosis Programme, Human Sciences Research Council, Cape Town, South Africa;; 10 Department of Medical Statistics, London School of Hygiene and Tropical Medicine, and; 11 Department of Medicine, Imperial College London, United Kingdom

**Keywords:** HIV-1, tuberculosis, immune reconstitution inflammatory syndrome, matrix metalloproteinase, procollagen III N-terminal propeptide

## Abstract

**Background:**

Extensive immunopathology occurs in human immunodeficiency virus (HIV)/tuberculosis (TB) coinfection, but the underlying molecular mechanisms are not well-defined. Excessive matrix metalloproteinase (MMP) activity is emerging as a key process but has not been systematically studied in HIV-associated TB.

**Methods:**

We performed a cross-sectional study of matrix turnover in HIV type 1 (HIV-1)–infected and –uninfected TB patients and controls, and a prospective cohort study of HIV-1–infected TB patients at risk of TB immune reconstitution inflammatory syndrome (TB-IRIS), in Cape Town, South Africa. Sputum and plasma MMP concentrations were quantified by Luminex, plasma procollagen III N-terminal propeptide (PIIINP) by enzyme-linked immunosorbent assay, and urinary lipoarabinomannan (LAM) by Alere Determine TB LAM assay. Peripheral blood mononuclear cells from healthy donors were cultured with *Mycobacterium tuberculosis* and extracellular matrix in a 3D model of TB granuloma formation.

**Results:**

MMP activity differed between HIV-1–infected and –uninfected TB patients and corresponded with specific TB clinical phenotypes. HIV-1–infected TB patients had reduced pulmonary MMP concentrations, associated with reduced cavitation, but increased plasma PIIINP, compared to HIV-1–uninfected TB patients. Elevated extrapulmonary extracellular matrix turnover was associated with TB-IRIS, both before and during TB-IRIS onset. The predominant collagenase was MMP-8, which was likely neutrophil derived and *M. tuberculosis*–antigen driven. *Mycobacterium tuberculosis*–induced matrix degradation was suppressed by the MMP inhibitor doxycycline in vitro.

**Conclusions:**

MMP activity in TB differs by HIV-1 status and compartment, and releases matrix degradation products. Matrix turnover in HIV-1–infected patients is increased before and during TB-IRIS, informing novel diagnostic strategies. MMP inhibition is a potential host-directed therapy strategy for prevention and treatment of TB-IRIS.

## Summary

Matrix metalloproteinase (MMP) activity in tuberculosis (TB) diverges according to HIV serostatus and compartment, releasing the matrix degradation product PIIINP into plasma. Plasma PIIINP and MMP-8 are potential predictive biomarkers of TB immune reconstitution inflammatory syndrome, whereas doxycycline inhibits TB-driven matrix degradation.

Tuberculosis (TB) and human immunodeficiency virus type 1 (HIV-1) infection are global pandemics with complex interplay. HIV-1 increases the risk of TB, while TB accelerates HIV-1 progression and is the commonest cause of HIV-related death [1]. Initiation of antiretroviral therapy (ART) reduces mortality, but is frequently complicated by development of tuberculosis immune reconstitution inflammatory syndrome (TB-IRIS) [2]. TB-IRIS is characterized by an acute inflammatory response to *Mycobacterium tuberculosis* (*Mtb*) presenting either in an uncharacteristically exaggerated form (unmasking IRIS), or as deterioration in a patient already receiving TB treatment (paradoxical IRIS) [3].

In immunocompetent adults, *Mtb* typically causes apical pulmonary disease with cavitation, which drives transmission and spread [4]. Conversely, in advanced HIV-1 infection, disseminated disease is more common and pulmonary cavitation less frequent [1, 5]. In paradoxical TB-IRIS, focal inflammatory pathology primarily affects the lung and lymph nodes, causing tissue damage [6]. Although specific features have been described, such as hypercytokinemia and inflammasome activation, the final effectors of this immunopathology are poorly defined [2, 6–9]. In HIV-uninfected TB patients, pulmonary immunopathology is driven by matrix metalloproteinases (MMPs), in particular the collagenase MMP-1, releasing matrix degradation products [10, 11]. Pulmonary MMPs are suppressed in advanced HIV-1 infection, providing a mechanism for reduced lung cavitation [12].

In this study, we systematically explored MMP activity and immunopathology in HIV-1–associated TB. We hypothesized that HIV-1–associated TB would be characterized by reduced MMP activity at TB diagnosis compared to HIV-uninfected TB, but that increased MMP activity would associate with inflammatory pathology during TB-IRIS. Our insights inform novel approaches to risk stratify and diagnose TB-IRIS, and also host-directed interventions to prevent pathology.

## MATERIALS AND METHODS

Full methods are provided in the Supplementary Data. The study was approved by the University of Cape Town Human Research Ethics Committee (REF 516/2011). Cross-sectional study participants were healthy volunteers, patients with symptoms requiring assessment, or patients recently diagnosed with TB (Supplementary Table 1). Longitudinal study participants were ART-naive HIV-1–infected patients with a CD4 count <200 cells/µL and recently diagnosed TB. Longitudinal study visits occurred at TB diagnosis (TB0), ART initiation (ARV0), and 2 (ARV2) and 4 (ARV4) weeks of ART. Induced sputum and venous blood were collected. TB-IRIS diagnosis was assigned retrospectively on case review, using International Network for the Study of HIV-associated IRIS (INSHI) criteria [3]. Chest radiographic inflammation (0–10) and sputum acid-fast bacilli (0–6) were scored as previously described [12].

### Laboratory Analyses

Sputum and plasma samples were analyzed by Bio-Rad Bio-Plex 200 using MMP beads (R&D Systems, Abingdon, United Kingdom). Procollagen III N-terminal propeptide (PIIINP) enzyme-linked immunosorbent assays (Cloud Clone Corp) and urine lipoarabinomannan (LAM) assays (Alere Determine TB LAM assay) were performed as per the manufacturers’ instructions.

### PBMC Stimulation With H37Rv

Cryopreserved peripheral blood mononuclear cells (PBMCs) from a separate cohort of 22 TB-IRIS patients and 22 non-IRIS controls were stimulated with heat-killed H37Rv *Mtb*, as previously described [13]. Culture supernatants were harvested at 24 hours and MMP-8 quantified by Luminex.

### Extracellular Matrix 3D Modeling

Alginate microspheres incorporating healthy donor PBMCs and collagen were generated by bioelectrospray methodology using a Nisco encapsulator [14]. Stimulated cells were preinfected with ultraviolet-killed *Mtb*. DQ-labeled fluorescent gelatin or collagen (Invitrogen) was incorporated into the microspheres to quantitate matrix destruction [15]. Doxycycline (10 μg/mL or 20 μg/mL) was added to media after microsphere generation.

### Statistical Analysis

Statistical analysis was performed using Prism 6 (GraphPad) and Stata version 14 software. Two-tailed Fisher exact or Mann-Whitney *U* test was performed for key comparisons. Correlations were assessed by Spearman rank-order correlation coefficients. Unadjusted and adjusted linear regression models were fitted to quantify effects and adjust for age, sex, and smoking status. Repeated-measures 2-way analysis of variance with Tukey posttest comparison compared time-points and conditions in the TB granuloma model.

## RESULTS

### Cross-sectional Study Participants

In the cross-sectional study, 227 participants were enrolled. Of these, 17 were excluded (unable to obtain samples, n = 8; diagnostic uncertainty, n = 9), leaving 210 for analysis (Supplementary Figure 1). Participant demographic and clinical characteristics are described in (Table 1). HIV-infected TB patients had a median CD4 count of 172 (interquartile range [IQR], 91–351) cells/µL. Age, sex, and body mass index (BMI) were similar in TB (HIV uninfected [HIV^−^]) and TB (HIV infected [HIV^+^]). However, smoking was more prevalent in TB (HIV^−^). TB (HIV^−^) and TB (HIV^+^) were associated with diverse pulmonary pathologies on chest radiograph. Frequency of cavities and median chest radiograph inflammation score were both reduced in TB (HIV^+^) compared with TB (HIV^−^). CD4 count and the number of cavities positively correlated (*r* = 0.357, *P* = .016), suggesting that destructive pulmonary pathology is reduced in advanced TB (HIV^+^). Microbiological confirmation of TB was similar for TB (HIV^−^) and TB (HIV^+^) (Supplementary Table 2). However, sputum smear positivity was more common in TB (HIV^−^).

**Table 1.** Demographics and Clinical Characteristics of Cross-sectional Study Participants

	HIV-Uninfected			HIV-Infected			*P* Value
Diagnostic Category	Healthy Control	Respiratory Symptomatic	TB Patient	Healthy Control	Respiratory Symptomatic	TB Patient	TB (HIV^−^) vs TB (HIV^+^)
Frequency, No.	61	24	39	16	17	53	NA
Median age, y (IQR)	28 (24–34)	29 (25–38)	37 (30–42)	33 (28–36)	33 (30–37)	32 (29–36)	.063
Female	19 (31)	5 (21)	13 (33)	11 (69)	10 (59)	25 (47)	.205
Smoking history, current or former	43 (70)	16 (67)	23 (59)	5 (31)	6 (35)	14 (26)	.003
Symptoms and examination findings							
Productive cough	0 (0)	6 (25)	31 (79)	0 (0)	5 (30)	30 (57)	.027
Nonproductive cough	0 (0)	6 (25)	10 (26)	0 (0)	8 (47)	21 (40)	.186
Hemoptysis	0 (0)	0 (0)	6 (15)	0 (0)	0 (0)	8 (15)	1.000
Night sweats	1 (2)	8 (33)	27 (69)	0 (0)	8 (47)	44 (83)	.138
Weight loss	6 (10)	5 (21)	20 (51)	3 (19)	4 (24)	30 (57)	.675
Median symptom duration, d (IQR)	NA	14 (5–75)	28 (14–30)	NA	30 (30–60)	30 (14–30)	.800
Median BMI	22.9 (21.2–26.4)	21.9 (19.9–24.2)	21.6 (20.1–25.8)	26.9 (22.8–32.9)	27 (20.4–31.8)	22.9 (20.4–25.3)	.493
Median temperature, °C (IQR)	36.1 (35.8–36.3)	35.9 (35.6–36.2)	36.2 (35.9–36.6)	36.1 (35.8–36.5)	35.9 (35.6–36.4)	36.6 (36.0–37.5)	.054
Median heart rate, beats/ min (IQR)	72 (68–78)	70 (66–78)	80 (68–88)	74 (70–82)	70 (64–83)	84 (77–98)	.009
Median systolic blood pressure, mm Hg	120 (119–130)	130 (120–133)	120 (110–120)	120 (112–128)	120 (111–130)	117 (110–120)	.617
Median diastolic blood pressure, mm Hg	80 (70–86)	80 (71–85)	80 (70–80)	80 (70–83)	80 (76–90)	75 (70–80)	.589
Median respiratory rate, breaths/min (IQR)	16 (16–18)	18 (16–20)	20 (16–24)	18 (16–20)	17 (16–20)	23 (18–28)	.074
Extrapulmonary TB features	NA	NA	8 (21)	NA	NA	26 (49)	.008
Laboratory parameters							
Median hemoglobin concentration, g/dL (IQR)	14.3 (13.3–15.0)	14.3 (13.8–15.3)	12.7 (12.2–13.7)	12.6 (11.8–13.7)	13.2 (12.7–13.9)	10.7 (9.4–12.0)	<.0001
Median total white cell count, ×10^9^/L (IQR)	6.06 (4.84–7.48)	5.81 (4.58–7.55)	7.45 (5.84–9.00)	4.95 (4.19–5.88)	5.24 (4.28–6.28)	5.48 (4.00–7.73)	.002
Median neutrophil count (IQR)	3.32 (2.39–4.53)	3.32 (2.21–4.88)	5.11 (3.4–6.71)	2.53 (1.9–3.59)	2.32 (1.9–3.45)	3.54 (2.33–5.34)	.009
Median neutrophil % (IQR)	55.2 (47.5–61.4)	55.5 (46.4–63.6)	65.8 (58.2–75.5)	51.8 (46.6–59.3)	51.4 (42.5–61.9)	68.9 (57.5–78.1)	.910
Median lymphocyte count (IQR)	1.99 (1.61–2.40)	1.8 (1.24–2.03)	1.57 (1.20–2.02)	1.53 (1.38–2.17)	1.81 (1.25–2.50)	1.08 (0.59–1.59)	.003
Median lymphocyte % (IQR)	32.7 (27.9–40.2)	32.6 (24.8–42.6)	21 (14.5–29.3)	32.3 (24.5–39.8)	34 (26.8–41.4)	20.8 (13.7–29.8)	.809
Median monocyte count (IQR)	0.35 (0.26–0.43)	0.36 (0.23–0.44)	0.55 (0.39–0.69)	0.37 (0.29–0.43)	0.27 (0.22–0.42)	0.34 (0.26–0.45)	<.0001
Median monocyte % (IQR)	5.2 (4.4–7.1)	5.6 (4.5–6.5)	6.6 (5.2–9.8)	6.9 (6.1–9.0)	5.9 (4.8–7.6)	6.8 (4.55–8.4)	.150
Median platelet count (IQR)	309 (247–355)	316 (262–380)	505 (355–579)	247 (182–327)	325 (263–377)	357 (249–464)	.001
Median C-reactive protein, mg/L (IQR)	1.3 (0.9–4.9)	3.3 (0.9–8.3)	46.9 (7.8–82.7)	1.4 (0.9–2.6)	2.7 (1.5–11.0)	57.1 (18.5–85.7)	.304
Median albumin concentration, g/dL (IQR)	47 (44–49)	47 (44–50)	40 (37–43)	43 (42–46)	44 (42–46)	34 (30–38)	<.0001
Median CD4 count, cells/µL (IQR)	NA	NA	NA	309 (194–409)	373 (151–526)	172 (91–351)	NA
Median CD4 % (IQR)	NA	NA	NA	15.8 (4.6–23.4)	18.5 (12.0–23.9)	12.7 (9.5–18.9)	NA
Radiographic features							
Median chest radiograph inflammation score (IQR)	0 (0–0)	1 (0–2)	6 (4–8)	0 (0–0)	1 (0–1)	4 (3–8)	.047
Cavities present on chest radiograph	0 (0)	0 (0)	27 (69)	0 (0)	0 (0)	15 (28)	<.001
Miliary TB	NA	NA	1 (3)	NA	NA	8 (16)	.071
Chest radiograph not available	5 (8)	1 (4)	1 (3)	0 (0)	1 (2)	4 (7)	.391

Data are presented as No. (%) unless otherwise indicated. *P* values are for Fisher exact or Mann-Whitney *U* test.

Abbreviations: BMI, body mass index; HIV, human immunodeficiency virus; IQR, interquartile range; NA, not applicable; TB, tuberculosis.

### Pulmonary MMP Profile Differs Between TB (HIV^−^) and TB (HIV^+^)

**Figure 1.**
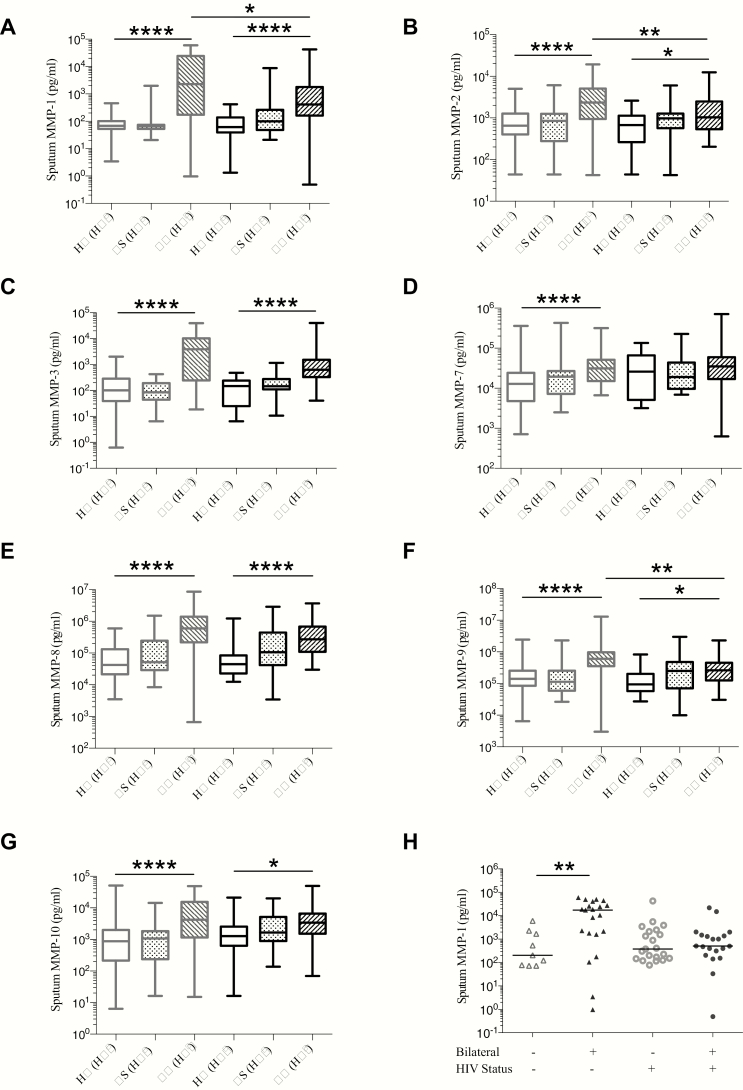
Pulmonary matrix metalloproteinase (MMP) concentrations are increased in tuberculosis (TB) and differ by human immunodeficiency virus (HIV) serostatus. Pulmonary TB is associated with increased MMP-1, -2, -3, -7, -8, -9, and -10 concentrations in sputum in comparison to respiratory symptomatic (RS) and healthy controls (HC) (*A–G*). Comparison of TB (HIV^−^) with TB (HIV^+^) demonstrated lower median sputum MMP-1 (*A*), -2 (*B*), -3 (*C*), and -9 (*F*) concentrations in TB (HIV^+^). Median MMP-8 was also reduced in TB (HIV^+^) compared to TB (HIV^−^), although to a lesser extent (*E*). TB (HIV^−^) patients with bilateral radiographic abnormalities had elevated sputum MMP-1, compared with TB (HIV^−^) patients with unilateral abnormalities (*H*). However, in TB (HIV^+^), MMP-1 was similar in patients with bilateral and unilateral chest radiographic involvement (*H*). In *A–G*, boxes represent the first and third quartiles and horizontal bars within indicate median values; whiskers indicate minimum and maximum values. In (*H*), triangles represent TB (HIV^−^) and circles represent TB (HIV^+^). Horizontal bars between the datasets indicate Mann-Whitney *U* test comparisons; in *A–G* comparisons between TB (HIV^−^) and HC (HIV^−^), TB (HIV^+^) and HC (HIV^+^), and TB (HIV^+^) and TB (HIV^−^) are shown. *P < .05, **P < .01, ****P < .0001.

In sputum, we found multiple MMPs to be elevated in TB patients compared to controls (Figure 1A–G). In TB (HIV^−^), median MMP-1 was increased 35-fold and 33-fold compared with HIV-1–uninfected respiratory symptomatics and healthy controls, respectively. However, in TB (HIV^+^), lower median sputum MMP-1, -2, -3, and -9 concentrations were observed compared with TB (HIV^−^) (Figure 1A–C and 1F). Unadjusted and adjusted linear regression modeling of the effect of HIV and TB infection on log-transformed sputum MMP concentrations provided further evidence of elevated sputum MMPs with TB, and reduced sputum MMPs in TB (HIV^+^) compared with TB (HIV^−^), after adjusting for age, sex, and smoking status (Supplementary Table 3). The greatest effect was for MMP-1 and MMP-3. We did not adjust for BMI as no association was observed with MMP concentrations. Sputum MMP concentrations by sex are reported in Supplementary Figure 2 and Supplementary Table 4.

We related radiographic features with MMP concentrations. Sputum MMP-1 positively correlated with cavity frequency in TB (HIV^−^) and TB (HIV^+^) (*r* = 0.592 and *r* = 0.533, respectively, both *P* < .0001). In TB (HIV^−^), the chest radiograph inflammation score positively correlated with sputum MMP-1 and MMP-3 (*r* = 0.452 and *r* = 0.453, both *P* = .011). However, in TB (HIV^+^), no such correlation was evident (Supplementary Table 5). In TB (HIV^−^) patients with bilateral chest radiograph lesions, median sputum MMP-1 was increased 85-fold compared to those with unilateral lesions. However, in TB (HIV^+^) patients with bilateral chest radiograph lesions, sputum MMP-1 was not increased compared to patients with unilateral lesions (Figure 1F).

In TB patients, sputum smear and culture positivity were associated with increased sputum MMP-1 and MMP-3, which were positively correlated with acid-fast bacilli score (Supplementary Figures 3 and 4). Together, these data support a role for sputum MMPs in pulmonary TB–driven matrix degradation. However, divergent MMP upregulation occurs in TB (HIV^−^) and TB (HIV^+^), with reduced sputum MMP-1 in TB (HIV^+^) associated with lesser pulmonary matrix destruction.

### Plasma PIIINP Is Elevated in TB and Further Increased in HIV-1–Associated TB

**Figure 2.**
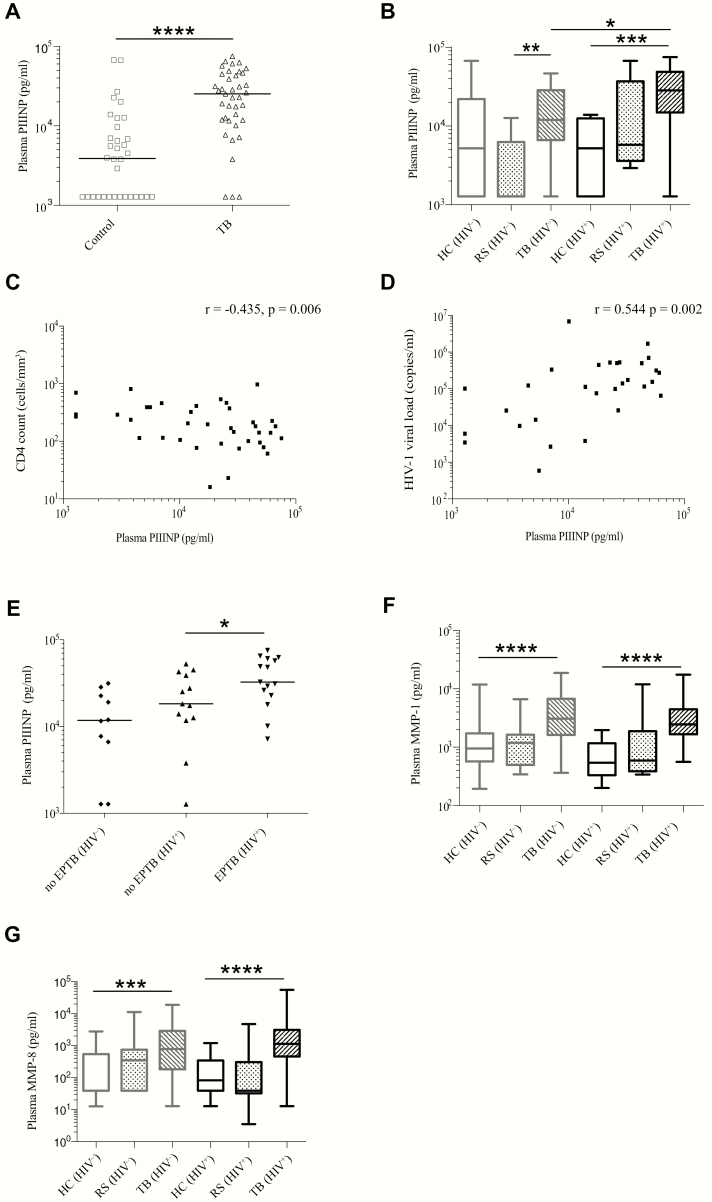
Tuberculosis (TB) increases systemic extracellular matrix turnover, which is further augmented by human immunodeficiency virus (HIV) coinfection. Plasma procollagen III N-terminal propeptide (PIIINP) concentration was elevated in TB patients compared to respiratory symptomatic (RS) and healthy controls (HC) (*A*). TB (HIV^+^) and TB (HIV^−^) patients had higher plasma PIIINP concentrations than corresponding controls (*B*). In contrast to sputum matrix metalloproteinases (MMPs), plasma PIIINP was further elevated in TB (HIV^+^) compared with TB (HIV^−^). In HIV^+^ patients, plasma PIIINP concentration and peripheral blood CD4 cell count negatively correlated (*C*), whereas HIV-1 viral load positively correlated with PIIINP concentration (*D*). Plasma PIIINP was elevated in patients with extrapulmonary TB (EPTB) compared to those without EPTB (*E*). Plasma MMP-1 (*F*) and plasma MMP-8 (*G*) were elevated in TB (HIV^−^) and TB (HIV^+^) compared to respective RS and HC, and did not differ by HIV serostatus in TB patients. Boxes represent the first and third quartiles and horizontal bars within indicate median values; whiskers indicate minimum and maximum values. Horizontal bars between the datasets indicate Mann-Whitney *U* test comparisons. *P < .05, **P < .01, ***P < .001, ****P < .0001. Correlations were performed using Spearman rank-order correlation coefficient.

MMP activity releases matrix degradation products such as PIIINP from type III collagen. Analysis of plasma in a subgroup of 73 patients of mixed HIV serostatus in the cross-sectional study (39 TB, 34 control) showed that PIIINP concentrations were elevated in TB patients compared to control patients (Figure 2A). Median PIIINP values were 25278 (IQR, 11787–45071) pg/mL and 3888 (IQR, 1278–10367) pg/mL, respectively (*P* < .0001). When analyzed according to HIV status, both TB (HIV^−^) and TB (HIV^+^) patients had higher plasma PIIINP concentrations than corresponding controls. However, we unexpectedly found that plasma PIIINP was further elevated in TB (HIV^+^) compared to TB (HIV^−^) (Figure 2B), despite the reduced sputum MMP concentrations. To investigate further, we related plasma PIIINP to clinical features. Plasma PIIINP negatively correlated with peripheral blood CD4 count (*r* = −0.435, *P* = .006; Figure 2C) and hemoglobin concentration (*r* = −0.557, *P* < .0001), and positively correlated with HIV-1 viral load (*r* = 0.544, *P* = .002; Figure 2D). Plasma PIIINP was significantly elevated in TB patients with extrapulmonary TB compared to those without (Figure 2E). Taken together with the sputum MMP analysis, this suggested that elevated plasma PIIINP in TB (HIV^+^) was due to increased extrapulmonary MMP activity.

### HIV Coinfection Does Not Suppress Systemic MMP Activity in TB

We therefore measured plasma MMP concentrations in the cross-sectional cohort (Figure 2F and 2G and Supplementary Figures 5 and 6). In TB (HIV^−^), MMP-1, MMP-7, and MMP-8 were elevated compared with HIV-uninfected controls, while plasma MMP-3, -9, and -10 were similar and MMP-2 was reduced. In TB (HIV^+^), the collagenases MMP-1 and MMP-8 were elevated in TB (HIV^+^) compared with HIV-infected controls (Figure 2F and 2G) and, in contrast to the findings in sputum, were not reduced compared to TB (HIV^−^).

### Paradoxical TB-IRIS Is Associated With Systemic Inflammation at TB Diagnosis

In the longitudinal cohort, 57 ART-naive TB patients with advanced HIV-1 (CD4 count <200 cells/µL) were enrolled (Supplementary Figure 1). Paradoxical TB-IRIS was diagnosed in 29 of 49 (59.2%) patients who completed follow-up. Of these, 25 met the INSHI criteria for TB-IRIS and 4 were probable TB-IRIS. Twenty patients did not develop TB-IRIS and were designated non-IRIS controls. Two non-IRIS controls were excluded (1 likely an elite controller having an undetectable HIV-1 viral load and therefore considered immunologically distinct, 1 developed hepatotoxicity on TB treatment delaying ART initiation), leaving 47 (29 TB-IRIS, 18 non-IRIS) patients in the final analysis.

Demographic and clinical characteristics of longitudinal study participants are reported in Supplementary Table 6. The median time to TB-IRIS symptom onset was 6 (IQR, 3.5–9.5; range, 1–23) days after ART initiation and patients presented with symptoms at a median of 14 (IQR, 9–15; range, 4–29) days. Clinical features of TB-IRIS presentations are reported in Supplementary Table 7. Predominant symptoms and signs were constitutional (n = 29 [100%]) and pulmonary (n = 27 [93.1%]). Patients with TB-IRIS were unwell: hospital admission was required in 13 (45%) TB-IRIS patients during study follow-up, compared with only 1 (6%) non-IRIS control (*P* = .007).

**Figure 3.**
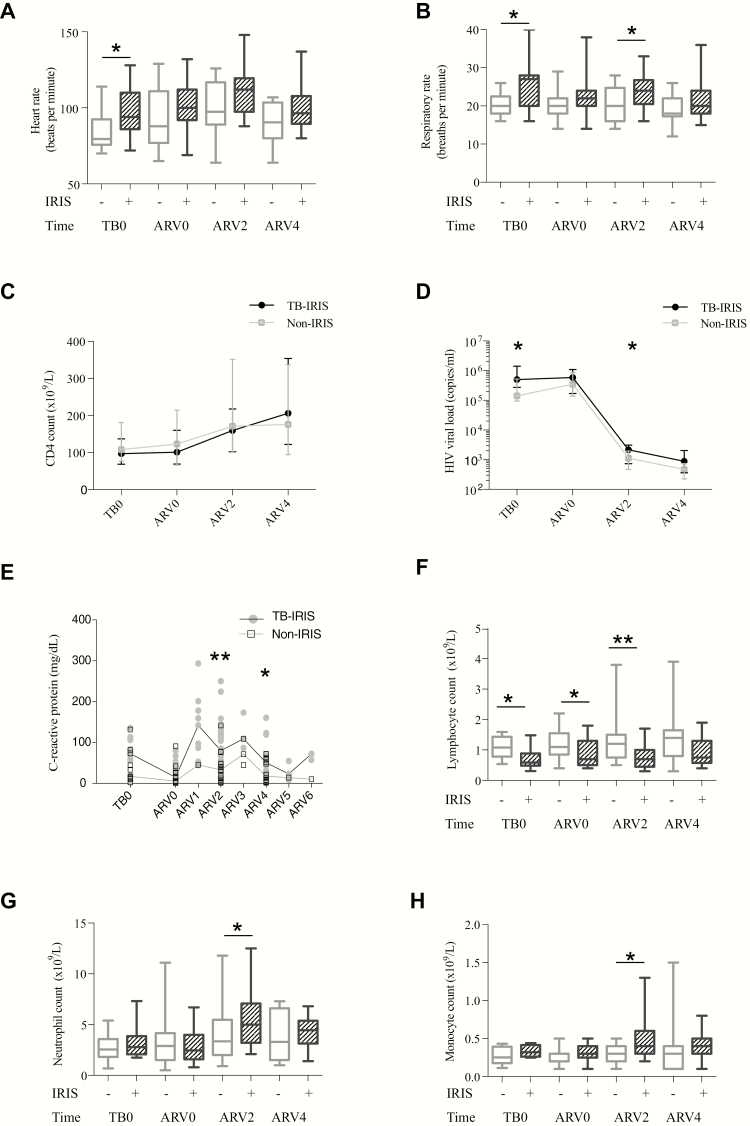
Paradoxical tuberculosis immune reconstitution inflammatory syndrome (TB-IRIS) is characterized by systemic inflammation at TB diagnosis and during TB-IRIS. We studied 47 antiretroviral therapy (ART)–naive TB patients with advanced human immunodeficiency virus (HIV; CD4 count <200 cells/µL) at enrollment, who underwent clinical observation at TB diagnosis (TB0) and biweekly for the first 4 weeks of ART (ARV0, ARV2, ARV4). TB-IRIS patients were characterized by elevated heart rate (*A*) and elevated respiratory rate (*B*), compared to non-IRIS controls. CD4 count increased in both TB-IRIS patients and non-IRIS controls following ART initiation (*C*) and concurrently HIV-1 viral load reduced (*D*), although median HIV-1 viral load was higher in TB-IRIS patients than in non-IRIS patients at TB diagnosis and at ARV2. Elevated C-reactive protein was a feature of TB-IRIS onset (*E*). Median lymphocyte counts were lower in TB-IRIS patients at all timepoints (*F*), whereas neutrophil counts (*G*) and monocyte counts (*H*) were increased at ARV2 and to a lesser extent at ARV4. In *A*, *B*, *F*, *G*, and *H*, boxes represent the first and third quartiles, horizontal bars within the median values, and whiskers the minimum and maximum values. In *C* and *D*, data are median values (TB-IRIS, filled circles; non-IRIS, open squares) linked by horizontal lines, and interquartile ranges are shown by vertical bars. In *E*, individual data points are shown, including results for unscheduled visits (ARV1, 3, 5, and 6 representing visits at 1, 3, 5 and 6 weeks of ART, respectively), and horizontal lines represent the median. In all panels, asterisks indicate Mann-Whitney *U* test comparisons; summary *P* values: *<.05 and **<.01.

Clinical signs that characterized TB-IRIS patients were elevated heart rate (Figure 3A) and respiratory rate (Figure 3B) at TB diagnosis, as well as at TB-IRIS presentation, compared to non-IRIS controls. In both TB-IRIS and non-IRIS patients, CD4 count increased in the first 2 weeks of ART (Figure 3C), and HIV-1 viral load reduced (Figure 3D), although median HIV-1 viral load was higher in TB-IRIS patients than in non-IRIS patients at TB diagnosis and at ARV2. Markedly increased C-reactive protein occurred at TB-IRIS presentation (Figure 3E). Median lymphocyte counts were lower in TB-IRIS patients at all timepoints (Figure 3F), whereas neutrophil counts (Figure 3G) and monocyte counts (Figure 3H) were increased at ARV2. Therefore, TB-IRIS was frequent, associated with significant morbidity and characterized by marked features of systemic inflammation at TB diagnosis, which partially resolved with TB treatment but recurred at the time of TB-IRIS.

### Immunopathology in Paradoxical TB-IRIS Is Associated With Increased MMP Activity

**Figure 4.**
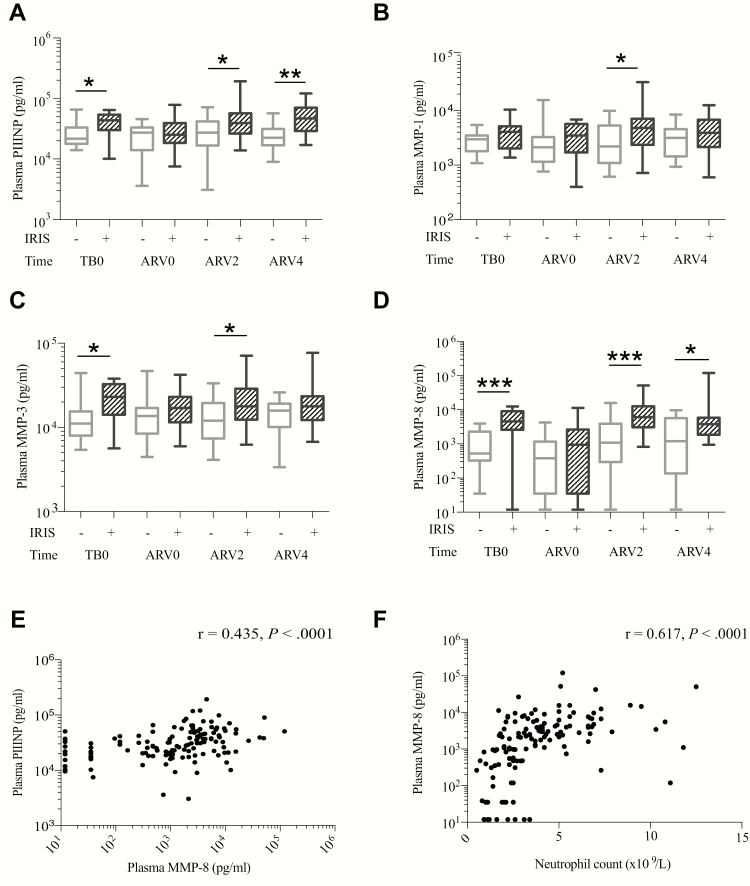
Immunopathology in paradoxical tuberculosis immune reconstitution inflammatory syndrome (TB-IRIS) is associated with increased matrix metalloproteinase (MMP) activity. Plasma procollagen III N-terminal propeptide (PIIINP) was elevated in TB-IRIS patients compared to non-IRIS control patients at the time of TB diagnosis and also at IRIS onset (*A*). Plasma MMPs were elevated in TB-IRIS compared to non-IRIS controls, including MMP-1 (*B*), MMP-3 (*C*), and, most consistently, MMP-8 (*D*). Plasma MMP-8 positively correlated with plasma PIIINP (*E*) and also with neutrophil count (*F*). In *A–D*, boxes represent the first and third quartiles; horizontal bars within median values and whiskers minimum and maximum values. Comparisons are by Mann-Whitney *U* test. *P < .05, **P < .01, ***P < .001. In *E* and *F*, individual data points are plotted by filled circles. Spearman rank-order correlation coefficient *r* and *P* values are reported for correlations.

To investigate the mechanism of immunopathology in TB-IRIS, we measured sputum MMPs and plasma PIIINP longitudinally. We observed no consistent association of increased sputum MMPs with TB-IRIS diagnosis (Supplementary Figure 7 and Supplementary Tables 8 and 9). In contrast, plasma PIIINP was elevated in TB-IRIS compared to non-IRIS patients both at the time of TB diagnosis, and during TB-IRIS (ARV2 and ARV4), but not at ART initiation (Figure 4A). At TB0, patients who later developed TB-IRIS had a median plasma PIIINP more than double that in non-IRIS controls: 43600 (IQR, 30021–63913) pg/mL vs 21651 (IQR, 17757–33196) pg/mL, respectively (*P* = .036).

To investigate the hypothesis that the elevated PIIINP resulted from systemic MMP activity in TB-IRIS, we examined plasma MMP concentrations. Plasma MMP-1, -3, and -8 were elevated in TB-IRIS compared to non-IRIS patients (Figure 4B–D and Supplementary Tables 8 and 10). MMP-8 (neutrophil collagenase) was the most significantly increased, in a similar pattern to PIIINP, suggesting that systemic collagenase activity caused matrix degradation and PIIINP production during TB-IRIS. Supporting this, MMP-8 correlated with plasma PIIINP concentration (*r* = 0.435, *P* < .0001) (Figure 4E). As neutrophils may be a source of MMP-8 and neutrophil counts were elevated in TB-IRIS patients, we assessed the correlation between plasma MMP-8 and neutrophil count and percentage. MMP-8 concentration positively correlated with neutrophil count (*r* = 0.617, *P* < .0001; Figure 4F) and percentage (*r* = 0.664, *P* < .0001).

### *Mtb* Antigen Is Associated With Elevated MMP Concentrations in TB-IRIS

**Figure 5.**
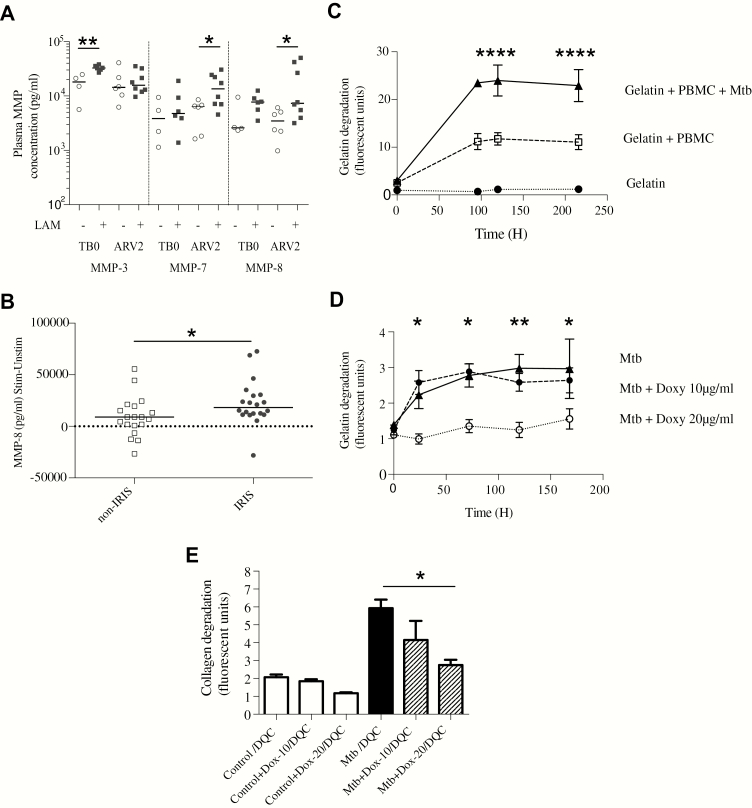
Elevated matrix metalloproteinases (MMPs) associate with increased tuberculosis (TB) antigen load, and *Mycobacterium tuberculosis* (*Mtb*)–driven MMP activity is inhibited by doxycycline (Doxy/Dox). Tuberculosis immune reconstitution inflammatory syndrome (TB-IRIS) patients with positive urinary lipoarabinomannan (LAM) had increased plasma MMP-3, MMP-7, and MMP-8 compared with TB-IRIS patients who were LAM negative (*A*). Plasma MMP-3 was higher at TB diagnosis (TB0) but not at 2 weeks of antiretroviral therapy (ARV2), whereas MMP-7 and MMP-8 were most significantly increased at ARV2. MMP-8 concentrations were measured in culture supernatants of peripheral blood mononuclear cells (PBMCs) stimulated with heat-killed H37Rv *Mtb* in a cohort of 22 TB-IRIS patients and 22 non-IRIS controls (*B*). After stimulation, MMP-8 secretion was greater from TB-IRIS PBMCs than non-IRIS controls. In a 3D cell culture model of TB, microspheres were impregnated with ultraviolet-killed *Mtb*-stimulated PBMCs and either DQ-gelatin or DQ-collagen (DQC), which increase in fluorescence when cleaved. *Mtb* stimulation increased total gelatin degradation within microspheres compared to control PBMCs (*C*). Addition of doxycycline to the surrounding cell culture media inhibited extracellular matrix breakdown (*D*). Similarly, doxycycline suppressed *Mtb*-driven collagen degradation in a dose-dependent manner (*E*). In *A* and *B*, horizontal lines indicate medians, and comparisons between groups are by Mann-Whitney *U* test analysis; in *C–E*, means and standard error of the mean are shown and analyses are by 2-way repeated measures analysis of variance, with Tukey posttest comparison. Comparisons shown are for gelatin + PBMC + *Mtb* compared to gelatin + PBMC (C) and doxycycline 20 μg/mL compared to *Mtb* alone. Summary *P* values: *<.05, **<.01, ****<.0001.

We further hypothesized that increased MMP activity in TB-IRIS patients was secondary to increased mycobacterial antigen load. The frequency of sputum smear positivity, culture positivity, smear score, and time to culture positivity was similar between TB-IRIS and non-IRIS patients. However, these indices represent *Mtb* antigen in the pulmonary compartment. We therefore measured urinary LAM, indicative of disseminated TB, in patients for whom a urine sample was available. In an adjusted regression analysis, IRIS was associated with increased odds of a positive urine LAM finding (odds ratio, 10.9 [95% confidence interval, 1.02–115.88], *P* = .048; Supplementary Table 11). In an analysis of TB-IRIS patients only, those LAM positive had higher plasma MMP-3, MMP-7, and MMP-8 than TB-IRIS patients who were LAM negative (Figure 5A).

We next examined the effect of antigen stimulation on MMP activity in TB-IRIS patients. We studied MMP-8 concentrations in PBMC culture supernatants from a previously published cohort of TB-IRIS and non-IRIS controls sampled at the time of TB-IRIS onset [13]. In TB-IRIS patients, MMP-8 secretion was increased following stimulation with heat-killed H37Rv *Mtb* compared to non-IRIS controls (Figure 5B).

### Doxycycline Suppresses *Mtb*-Driven Matrix Degradation

Doxycycline is a licensed MMP inhibitor and reduces collagenase activity. We studied the inhibitory effect of doxycycline in a 3D cell culture model of TB that recapitulates key components of human granuloma formation using a functional readout of matrix destruction [15]. Stimulation of PBMCs with ultraviolet-killed *Mtb* increased degradation of gelatin within microspheres over time compared to uninfected cells (Figure 5C). Doxycycline in cell culture media around microspheres inhibited this breakdown (Figure 5D). Similarly, doxycycline suppressed *Mtb*-driven collagen degradation in a dose-dependent manner (Figure 5E).

## DISCUSSION

Despite some advances, treatment of TB remains a great challenge, due to lengthy regimens, poor side-effect profiles, drug interactions, and drug resistance [16]. These problems are further compounded by HIV-1 coinfection [17]. Host-directed therapies have been proposed as a novel strategy to improve treatment outcome, but their development requires greater understanding of pathological and protective immune responses [16, 18]. In this study, we identified key differences between MMPs that cause immunopathology in TB dependent on HIV-1 status and characterized MMPs in TB-IRIS. In HIV-uninfected TB patients, MMP activity was prominent in the pulmonary compartment and MMP-1 was dominant, whereas in HIV-1–infected patients, higher plasma PIIINP may represent MMP-driven tissue destruction at extrapulmonary sites, with MMP-8 as the principal protease. We identified PIIINP as a pathological marker of excessive MMP activity at TB diagnosis and during TB-IRIS, with potential to risk stratify individuals prior to ART and also to diagnose TB-IRIS. In addition, the MMP inhibitor doxycycline has potential as a host-directed therapy to prevent TB-IRIS.

Our findings of increased sputum MMPs in TB, and reduced sputum MMPs in HIV-1-associated TB, concur with findings in previous smaller studies [10, 12]. We previously reported that multiple MMP genes were upregulated in TB-IRIS PBMCs restimulated with heat-killed H37Rv *Mtb* [13]. MMP-8 was not among the upregulated genes. However, while most MMPs are regulated at the transcriptional level, MMP-8 is predominantly presynthesized in neutrophils and therefore may not be identified by gene expression analysis. We have previously reported compartmentalized inflammatory responses in HIV-infected patients with tuberculous meningitis (TBM), in cerebrospinal fluid (CSF) and plasma, with elevated CSF MMP-1, -7, and -10 in TBM-IRIS compared with non-IRIS controls, although MMP-8 was not studied [19]. Ravimohan et al found that an increase in plasma MMP-8 at week 4 of ART relative to baseline pre-ART levels was associated with increased TB-IRIS risk and abnormal pulmonary function tests after TB treatment completion, consistent with our finding that MMP-8 is a key collagenase in TB-IRIS [20]. In addition, we have previously demonstrated that neutrophils were an important source of MMP-8 in TB and that neutrophilia was associated with poor outcomes [21, 22].

Dysregulated innate immune responses have been implicated in TB-IRIS pathophysiology [6, 7, 9, 23]. Elevated innate proinflammatory cytokines, monocyte activation, cytotoxicity, and inflammasome activation have been associated with TB-IRIS, implying global activation of the innate immune response [6, 8, 9, 24, 25], but these studies do not identify the ultimate effectors of tissue destruction. An association between TB-IRIS and mycobacterial antigen load has been demonstrated [9, 26]. Our results suggest that either *Mtb* antigen leads to increased MMP activity or that increased MMP activity in patients who develop TB-IRIS causes increased extracellular matrix destruction, thereby increasing detection of urinary antigen. In HIV-1–infected TB patients on ART, elevated hyaluronic acid, a glycosaminoglycan component of the extracellular matrix, was associated with poor outcomes including death [25]. Therefore, matrix degradation products may have a prognostic role in HIV-1–associated TB and indicate high TB-IRIS risk.

Currently, the only established immunomodulatory strategy for TB or TB-IRIS is corticosteroid therapy, used adjunctively in central nervous system and pericardial TB, and in treatment of paradoxical TB-IRIS [27]. Corticosteroids suppress *Mtb*-driven MMPs and this may contribute to their beneficial effects [28–30]. Our 3D model of TB granuloma formation demonstrates the potential of doxycycline to inhibit *Mtb*-driven matrix degradation. Extracellular matrix integrity favors host cell survival in *Mtb* infection, and therefore matrix-protective strategies may improve outcome in TB without exacerbating HIV-1–related immune compromise [31]. Doxycycline, a licensed MMP inhibitor, is cheap, safe, and widely available and could be studied as an immunomodulatory adjuvant in TB treatment, with the added benefit of bacteriostatic antimycobacterial activity [12, 32].

We report a large study investigating mechanisms of immunopathology in TB. However, as an observational study, we cannot attribute causality, nor exclude the possibility that unmeasured confounding factors contributed to measured associations. We adjusted for sex and smoking status, factors that have been associated with divergent MMP responses, but cannot exclude alternative confounders [33, 34]. The incidence of TB-IRIS (59%) was high, causing significant morbidity. Sputum samples were not available from some severely unwell TB-IRIS patients who were unable to expectorate, which may have resulted in an underestimation of effect in the longitudinal sputum analysis.

In summary, our work supports a central role for MMPs in causing tissue damage in TB and TB-IRIS, generating matrix degradation products. Differential MMP expression and compartmentalization occurs in HIV-1–infected patients. Systemic MMP-8 is the dominant protease in TB-IRIS, in contrast to pulmonary-localized MMP-1 in HIV-uninfected TB patients. Matrix degradation products are promising biomarkers of TB-IRIS risk prior to and during clinical onset. Doxycycline, an MMP inhibitor, may prevent immunopathology in TB-IRIS.

## Supplementary Data

Supplementary materials are available at *Clinical Infectious Diseases* online. Consisting of data provided by the authors to benefit the reader, the posted materials are not copyedited and are the sole responsibility of the authors, so questions or comments should be addressed to the corresponding author.

## Supplementary Material

Supplementary Information

## Notes

***Author contributions.*** N. F. W., K. A. W., G. M., R. J. W., J. S. F., and P. T. E. conceived and designed the clinical study. N. F. W., G. M., R. G., J. M. P., and R. J. W. recruited the clinical cohort. N. F. W., K. A. W., and A. C. performed laboratory analyses of cross-sectional and longitudinal study samples. K. A. W., G. M., R. T., and R. J. W. conceived and performed TB-IRIS PBMC *Mtb* stimulation experiments, and L. B. T. and P. T. E. conceived and performed the 3D TB granuloma experiments. N. W. and C. O. performed analysis of data. N. F. W. and P. T. E. hold all primary data and are responsible for the integrity of the data. All authors contributed to the writing of the manuscript and approved the final submitted version.

***Acknowledgments.*** We are grateful to the Clinical Infectious Diseases Research Initiative clinical research team and Tara Sathyamoorthy for assistance with patient recruitment; and to the participants, staff, and patients of Ubuntu Clinic and the Western Cape Government: Health.

***Disclaimer.*** The funders had no role in the study design, data collection, data analysis, data interpretation, or writing of this report. The opinions, findings, and conclusions expressed in this manuscript reflect those of the authors alone.

***Financial support.*** This work was supported by the Wellcome Trust (award numbers 203135, 094000, 104803, 098316); UK National Institute for Health Research (NIHR); Imperial College NIHR Biomedical Research Centre; the Francis Crick Institute which receives its core funding from Cancer Research UK (FC00110218), the UK Medical Research Council (FC00110218), and the Wellcome Trust (FC00110218); MRC-UK; UK National Centre for the 3Rs NC/L001039/1; British Federation of Women Graduates (Ruth Bowden Scholarship to N. F. W.); European Union (FP7 HEALTH-F3-2012-305578); US National Institutes of Health (award number R33AI102239); National Research Foundation (NRF) of South Africa (award numbers 96841 and 64787) and NRF incentive funding (UID: 85858); David and Elaine Potter Foundation; the South African Research Chairs Initiative of the Department of Science and Technology; and the South African Medical Research Council through its TB and HIV Collaborating Centres Programme, with funds received from the National Department of Health (RFA number SAMRC-RFA-CC: TB/HIV/AIDS-01-2014) and the National Health Scholars Programme.

***Potential conflicts of interest.*** All authors: No reported conflicts of interest. All authors have submitted the ICMJE Form for Disclosure of Potential Conflicts of Interest. Conflicts that the editors consider relevant to the content of the manuscript have been disclosed.
