# Supplementary material for: Matrix Degradation in Human Immunodeficiency Virus Type 1–Associated Tuberculosis and Tuberculosis Immune Reconstitution Inflammatory Syndrome: A Prospective Observational Study
Source: Clin Infect Dis. 2017 May 5;65(1):121–32. doi: 10.1093/cid/cix231 (PMC5815569; doi:10.1093/cid/cix231)
Supplement: Supplementary Information [file cix231_suppl_supplementary_information.pdf]

Matrix degradation in HIV-1-associated tuberculosis and tuberculosis immune reconstitution inflammatory syndrome: a prospective, observational study

Walker NF, Wilkinson KA, Meintjes G, Tezera L, Goliath R, Peyper JM, Tadokera R, Opondo C, Coussens AK, Wilkinson RJ, Friedland JS, Elkington PT.

Supplementary Information

Methods

Participant recruitment

The study was approved by the University of Cape Town (UCT) Human Research Ethics Committee (HREC REF 516/2011). Enrolment took place between 1st May 2012 and 1st December 2013. All participants provided informed consent prior to enrolment. Participants were recruited at Ubuntu HIV/TB clinic, Site B Khayelitsha and in the community. Clinic staff and patients were informed about the study by posters, presentations and informal discussions. Study participants were identified from patients routinely attending for assessment and/or treatment at Ubuntu clinic or at Khayelitsha Day Hospital, an outpatient facility on the same site. Written informed consent was obtained for all participants. Participants were recruited into the cross-sectional and / or the longitudinal study, based on study specific eligibility criteria. Pregnancy, age less than 18 years, asthma/chronic obstructive pulmonary disease, current anti-retroviral therapy (ART), confirmed multi-drug resistant tuberculosis and an inability to provide informed consent were exclusion criteria.

Cross-sectional study participants were healthy volunteers, patients with symptoms requiring assessment and recently diagnosed TB patients starting anti-tuberculosis therapy. HIV-infected patients were ART naive at enrollment. Cross-sectional study participants who were on anti-tuberculosis therapy were required to have had less than 3 doses prior to study samples being collected. Active TB was diagnosed on the basis of smear or culture positivity, or in cases of smear-negative TB according to international guidelines (for case definitions see Supplementary Table S1).[1,2] Once enrolled, cross-sectional study participants were provided appropriate follow up by the research team for study clinical results and were then followed up routinely by clinic staff, unless also eligible for the longitudinal study. Designation of study clinical categories was made retrospectively, following the results of all relevant investigations. Cross-sectional study participants who had symptoms but did not meet the criteria for TB diagnosis after all investigation results became available were designated respiratory symptomatics. Cross-sectional study participants who met eligibility criteria for the longitudinal study were co-

enrolled, or if eligibility became apparent after enrolment, were invited to subsequently enroll into the longitudinal study.

Longitudinal study participants were ART naïve HIV-infected patients with a low CD4 count (<200 cells/mm^3) and a recent diagnosis of TB. Following anti-tuberculosis therapy initiation and enrolment into the longitudinal study (study visit TB0) patients received counseling for ART initiation. Anti-tuberculosis therapy and ART followed national guidelines.[3,4] First-line ART at the time of the study was principally tenofovir, lamivudine, and efavirenz. ART was initiated typically two weeks after anti-tuberculosis therapy (study visit ARV0) and patients attended further scheduled study visits at two weeks post-ART initiation (ARV2, Day 14 +/- 72 hours) and four weeks post-ART initiation (ARV4, Day 28 +/-72 hours) for clinical assessment and sampling. Additionally, patients were requested to attend for assessment if any new symptoms or clinical deterioration occurred (study interim assessment) and were followed up to twelve weeks post-ART initiation. Clinical research staff telephoned participants regularly to reinforce this, to remind patients about scheduled visits and to investigate non-attendance. If a case of TB-IRIS was suspected, study samples were collected as at a scheduled visit, in addition to clinically indicated diagnostic tests. Where possible, when patients were hospitalized at the time of a study visit, they were visited by the study team for data and sample collection. Retrospective designation into one of three longitudinal study categories (paradoxical TB-IRIS (INSHI IRIS), probable paradoxical TB-IRIS not meeting INSHI criteria (non-INSHI IRIS / probable TB-IRIS), and no paradoxical TB-IRIS (non-IRIS)) followed the results of all relevant investigations and clinical follow up, and was made on case review by a consensus panel (comprising the study clinician NFW, and two clinical specialists: GM, RJW).

In both cross-sectional and longitudinal studies, demographic information was recorded at enrolment, including gender and smoking status. At each study visit, symptoms and clinical examination, full blood count, albumin, C-reactive protein (CRP) and chest radiograph were performed, plus additional investigations if clinically indicated. Induced sputum and venous blood were collected (see below) at each visit for microbiological and laboratory analysis.

Clinical Scoring System

A chest radiograph scoring system to quantify the extent of inflammation was used as previously described, excluding patients with miliary TB.[5] Each lung field was divided into 5 areas of equal size and a score of 0-10 was assigned based on the number of areas in which inflammatory pathology was visible. Cavities were recorded as present or absent and counted. The result of sputum microscopy following auramine staining was used to derive a sputum AFB score (0-6). Sputum AFB scores were assigned as follows: 0 = no AFB seen; 1 = scanty (1-9 per 100 immersion fields) AFB seen; 2 = 1+ AFB seen (10-99 per 100 immersion fields); 4 = 2+ AFB seen (1-10 per immersion field); 6 = 3+ AFB seen (≥10 per immersion field).

Sample collection and processing

Sputum induction was performed with 5% saline nebulized in 5-minute cycles, up to 20 minutes as tolerated. Induced sputum was not obtained if patients failed induction, refused induction or were at risk of an adverse event from induction due to respiratory distress, pneumothorax or history of asthma. Sputum was expectorated into two to three sterile collection containers. One or two sputum samples were sent for microbiological examination (smear microscopy and culture) by the National Health Laboratory Service (NHLS). Induced sputum for luminex analysis was transported on ice to UCT and stored at -80^{0}C, within 2 hours of collection. Samples were thawed in batches. Mucolysis was performed by adding 0.1% dithiothreitol and agitating for 20 minutes. Samples were then centrifuged and sterile filtered through a 0.2-mm Durapore membrane (Millipore, Watford, UK) prior to aliquoting into Sarstedt containers and storing at -80^{0}C.[6]

Venous blood was collected in sodium heparin vacutainers. Blood for luminex analysis and ELISA was transported on ice to UCT and centrifuged at 3500 rpm (2800g) for 10 minutes at 4^{0}C, within two hours of collection. The supernatant was aliquoted and stored at -80^{0}C in Sarstedt containers. Venous blood

for PBMC isolation was transported at room temperature to UCT, and processed within four hours of collection. PBMC were isolated by density gradient centrifugation over Ficoll and cryopreserved in heat-inactivated fetal calf serum (FCS) with 10% dimethyl sulfoxide (DMSO).

The NHLS processed blood samples for clinical laboratory analyses. Blood for full blood count and differential was collected in K2 EDTA tubes, transported at room temperature and measured using a Siemens Advia 2120I. Similarly, for HIV-infected patients, CD4 count was measured on a Beckman Coulter FC500MP. Serum was collected for albumin and C-reactive protein quantification on a Roche Modular, and for HIV-infected patients, HIV-1 viral load was measured on an Abbott M2000.

Urine samples were collected into sterile containers at each visit and transported to UCT laboratory on ice. Aliquots were stored at -80^{0}C.

MMP quantification

Samples were analyzed on the Bio-Rad Bio-Plex 200 System using MMP beads (R&D Systems, Abingdon, UK) and analyzed as per manufacturer's instructions. The sensitivity of the assays were: MMP-1 = 1.1 pg/ml, MMP-2 = 12.6 pg/ml, MMP-3 = 7.3 pg/ml, MMP-7 = 6.6 pg/ml, MMP-8 = 16.6 pg/ml, MMP-9 = 13.7 pg/ml, MMP-10 = 3.2 pg/ml. Induced sputum samples were diluted 1:5 or 1:50 in assay diluent. Plasma samples were run neat.

Procollagen III N-terminal Propeptide (PIIINP) ELISA

PIIINP ELISAs (Cloud Clone Corp, USA) were performed as per manufacturer's instructions on plasma samples from a subgroup of the cross-sectional study (73 consecutively recruited patients) and on the entire longitudinal cohort. Plasma samples were diluted 1:100 in phosphate-buffered saline (PBS). Positive controls for the assays were reconstituted lyophilized standard, while negative controls were standard diluent alone. The optical density was measured on an ELISA plate reader at 450nm. The

sensitivity of the assay was 25.6 pg/mL

Urine lipoarabinomannan (LAM) analysis

Urine LAM assays were performed on all available urine samples by the Department of Medical Microbiology, UCT, using Alere Determine TB LAM point-of-care assay in urine (7D2740). Scores 0-4 were assigned, as per manufacturer's instructions. Positive results were indicated by a score of 1-4.

PBMC stimulation with heat-killed H37Rv

Cryopreserved peripheral blood mononuclear cells (PBMC) from a separate cohort of 22 TB-IRIS patients and 22 non-IRIS controls were stimulated with heat-killed H37Rv Mtb as previously described.[7,8] Culture supernatants were harvested at 24 hours and MMP-8 quantified by luminex.

Extracellular matrix 3-D modelling

Microspheres were formed using bioelectrospray methodology as previously described.[9,10] PBMC stimulated with ultraviolet-killed Mtb were re-suspended in a solution of 3% Alginate (Pronova UP MVG alginate, Nova Matrix, Norway), 1 mg/ml of bovine gelatin solution (Sigma-Aldrich, UK) and 100 µg/ml of DQ Gelatin from pig skin (Invitrogen, Paisley, UK) in NaOH, HEPES and 7.5% $NaHCO_3$. Collagen spheres were made with human collagen (Advanced Biomatrix, Carlsbad, CA) and DQ collagen (Invitrogen, Paisley, UK). Microspheres were then placed in serum free medium and incubated at 37°C. Doxycycline, licensed for use as an MMP inhibitor, was then was added to media of specified wells (10 µg/ml or 20 µg/ml). Florescence was read sequentially using the GloMax Discover 96 well plate reader (Promega) at absorption maxima at 495 nm and fluorescence emission maxima at 515 nm. Microspheres formed from uninfected PBMC were used as controls.

Sample size and statistical analysis

Using the standard deviation from preliminary data, a sample size of 40 patients in each group in the cross-sectional study was expected to give an 80% power to detect 1.5-fold change in sputum MMP concentration between TB (HIV-) and TB (HIV+), with a two sided alpha of 0.05. As the longitudinal study was exploratory, and preliminary data was not available for a power calculation, the sample size was pragmatic, with a target of 32 TB-IRIS patients, and an expected TB-IRIS incidence of 30%.

Anonymised data was recorded in an Access database and exported to an Excel (Microsoft) spreadsheet. Statistical analysis was performed using Prism 6 (GraphPad, UK) and STATA 14. In the cross-sectional study, pre-specified comparisons were: TB (HIV-) and RS (HIV-), TB (HIV-) and HC (HIV-), TB (HIV+) and RS (HIV+), TB (HIV+) and HC (HIV+), TB (HIV+) and TB (HIV-). The key comparisons to address the primary hypotheses were TB (HIV-) and HC (HIV-), TB (HIV+) and HC (HIV+), and TB (HIV+) and TB (HIV-), and p values by Mann-Whitney U analysis for these comparisons are summarized on graphs using a bar and asterisks: *<0.05, ** <0.01, ***<0.001, ****<0.0001. Where p >0.05 for any of these three key comparisons, no asterisks are shown. In the longitudinal study pre-specified comparisons were between TB-IRIS (INSHI-IRIS and non-INSHI IRIS) and non-IRIS controls at each timepoint (TB0, ARV0, ARV2, ARV4).

In the cross-sectional analysis, unadjusted and adjusted linear regression models for the effect of HIV and TB infection on log-transformed MMP and PIIINP concentrations were fitted to quantify effects and adjust for potential confounding by age, sex and smoking status. We have examined both the log-transformed measures and the residuals from the model for normality and are satisfied that this assumption is not violated.

For the longitudinal data, unadjusted and adjusted hierarchical linear regression models for the effect of IRIS on log-transformed MMP and PIIINP concentrations were fitted, which also adjust for

clustering of repeated observations over time within individual. As in the models for the cross-sectional data, adjustment was made for age, sex and smoking status.

Further comparisons were performed to investigate specific hypotheses generated by these results. Two-tailed Fisher's Exact or Mann–Whitney U analysis was performed where two categorical or continuous variables were compared respectively. Hypothesis-driven correlations were assessed by Spearman rank-order correlation coefficients. Where patients missed study visits or were unable to donate samples, missing values were not imputed. Repeated measures two-way ANOVA with Tukey's post-test comparison was used to compare time-points and conditions in the TB granuloma model. A significance threshold was not set.

References

1. Siddiqi K, Lambert ML, Walley J. Clinical diagnosis of smear-negative pulmonary tuberculosis in low-income countries: the current evidence. *Lancet Infect Dis* 2003; **3**(5): 288-96.

2. WHO. Improving the diagnosis and treatment of smear-negative pulmonary and extra-pulmonary tuberculosis among adults and adolescents: recommendations for HIV-prevalent and resource-constrained settings. Geneva, 2006.

3. The South African Antiretroviral Treatment Guidelines 2013: Department of Health, Republic of South Africa, 2013.

4. National Tuberculosis Management Guidelines 2014: Department of Health, Republic of South Africa.

5. Walker NF, Clark SO, Oni T, et al. Doxycycline and HIV infection suppress tuberculosis-induced matrix metalloproteinases. *Am J Respir Crit Care Med* 2012; **185**(9): 989-97.

6. Elkington PT, Green JA, Friedland JS. Filter sterilization of highly infectious samples to prevent false negative analysis of matrix metalloproteinase activity. *J Immunol Methods* 2006; **309**(1-2): 115-9.

7. Tadokera R, Meintjes G, Skolimowska KH, et al. Hypercytokinaemia accompanies HIV-tuberculosis immune reconstitution inflammatory syndrome. *Eur Respir J* 2011; **37**(5): 1248-59.

8. Tadokera R, Meintjes GA, Wilkinson KA, et al. Matrix metalloproteinases and tissue damage in HIV-tuberculosis immune reconstitution inflammatory syndrome. *Eur J Immunol* 2014; **44**(1): 127-36.

9. Workman VL, Tezera LB, Elkington PT, Jayasinghe SN. Controlled Generation of Microspheres Incorporating Extracellular Matrix Fibrils for Three-Dimensional Cell Culture. *Adv Funct Mater* 2014; **24**(18): 2648-57.

10. Tezera LB, Bielecka MK, Chancellor A, et al. Dissection of the host-pathogen interaction in human tuberculosis using a bioengineered 3-dimensional model. *Elife* 2017; **6** pii: e21283.

.

Supplementary Figure Legends

Supplementary Figure S1 Tissue destruction in tuberculosis 2 (TDTB2) study patient enrolment

Screening took place in Ubuntu Clinic, Site B Khayelitsha. Following screening for exclusion criteria, 227 eligible patients were enrolled into the TDTB2 cross-sectional (CX) study. Subsequently, 17 were excluded from the analysis (unable to obtain samples n=8, diagnostic uncertainty n=9), leaving 210 patients remaining in the cross-sectional analysis. Fifty-seven participants were enrolled into the longitudinal (LS) study. Of these, 22 were HIV-infected TB patients with a CD4 count <200 cells/mm^3 who had been included in the cross-sectional study and 35 were HIV-infected patients with active TB and CD4 counts <200 cells/mm^3 who had received more than 3 doses of TB treatment at time of sample collection and so were enrolled exclusively into the longitudinal study. Eight patients were excluded from the analysis as they were lost to follow up and therefore could not be assigned as TB-IRIS or non-IRIS. Additionally, two patients in the non-IRIS control group were excluded following IRIS designation: one was likely an elite controller having an undetectable HIV-1 viral load throughout and therefore considered immunologically distinct, and one developed hepatotoxicity due to TB treatment delaying ART initiation. HC = Healthy control, RS = non-TB respiratory symptomatic, TB = tuberculosis patient, INSHI = International Network for the Study of HIV-associated IRIS.

Supplementary Figure S2 Sputum MMP concentrations by sex

Sputum MMP concentrations were measured in cross-sectional study participants by luminex and are reported for the 6 clinical categories by sex: Female (F) and Male (M). HC = Healthy control, RS = non-TB respiratory symptomatic, TB = tuberculosis patient.

Supplementary Figure S3 Sputum MMP concentrations by smear and culture status in TB patients

Sputum MMP concentrations in TB patients are reported by HIV-1 status and sputum smear (A) and culture (B) status. A trend towards elevated MMPs in smear positive and culture positive patients within HIV status groups was observed and Mann-Whitney U analysis was performed to assess the strength of evidence, demonstrating strong evidence for an association between smear and culture positivity and elevated sputum MMP-1 and -3, both in HIV-uninfected and –infected TB patients. Boxes represent the 1st and 3rd quartiles and horizontal bars within indicate median values, whiskers indicate minimum and maximum values. Horizontal bars between the datasets indicate Mann-Whitney U comparisons. P values are summarized by asterisks: *<0.05, **<0.01, ***<0.001, ****<0.0001.

Supplementary Figure S4 Sputum MMP-1 and -3 associate with smear positivity in TB

Sputum MMP-1 and MMP-3 positively correlate with sputum acid fast bacilli (AFB) score in TB patients. Correlation was assessed by Spearman rank-order correlation coefficient. Sputum AFB scores were assigned as follows: 0 = no AFB seen; 1 = scanty (1-9 per 100 immersion fields) AFB seen; 2 = 1+ AFB seen (10-99 per 100 immersion fields); 4 = 2+ AFB seen (1-10 per immersion field); 6 = 3+ AFB seen (≥10 per immersion field).

Supplementary Figure S5 Plasma MMP concentrations in the cross-sectional cohort

Plasma MMP concentrations were measured in cross-sectional study participants by luminex. Plasma MMP-7 (in addition to MMP-1 and MMP-8 shown in Figure 2) was elevated in TB (HIV-) compared to HIV-uninfected respiratory symptomatic (RS) and healthy control (HC) participants (A). In contrast, plasma MMP-2 was reduced in TB (HIV-) compared to HIV-uninfected RS and HC. MMP-3, -9 and -10 were similar in TB patients and controls. Boxes represent the 1st and 3rd quartiles and horizontal bars within indicate median values, whiskers indicate minimum and maximum values. Horizontal bars between the datasets indicate Mann-Whitney U comparisons. P values are indicated by asterisks: ****<0.0001.

Supplementary Figure S6 Plasma MMP concentrations by sex

Plasma MMP concentrations were measured in cross-sectional study participants by luminex and are reported for the 6 clinical categories by sex: Female (F) and Male (M). HC = Healthy control, RS = non-TB respiratory symptomatic, TB = tuberculosis patient.

Supplementary Figure S7 Induced sputum MMP concentrations in the longitudinal cohort

Induced sputum matrix metalloproteinase concentrations were measured by luminex in longitudinal study participants. There was no consistent elevation of MMP concentrations in TB-IRIS patients compared to non-IRIS controls. There was a trend towards elevated median MMP-7 and -10, which was most marked for MMP-7, at ARV0. Box and whisker plots are shown. Boxes represent the 1st and 3rd quartiles and horizontal bars indicate median values, whiskers indicate minimum and maximum values. TB-IRIS (shaded boxes) and non-IRIS controls (unshaded boxes) were compared at each timepoint by Mann-Whitney U analysis: *$p<0.05$.

Supplementary Figures

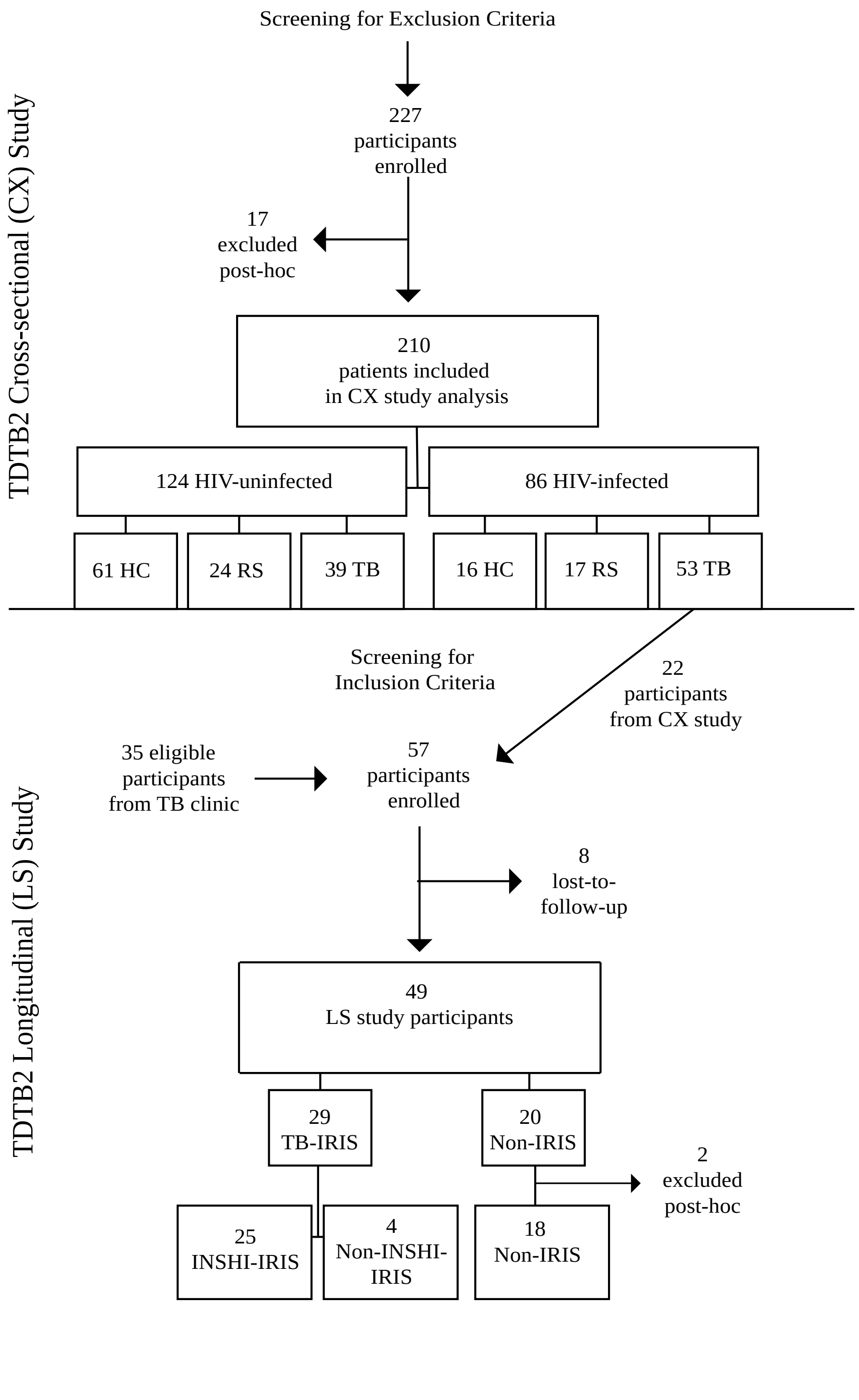

Supplementary Figure S1

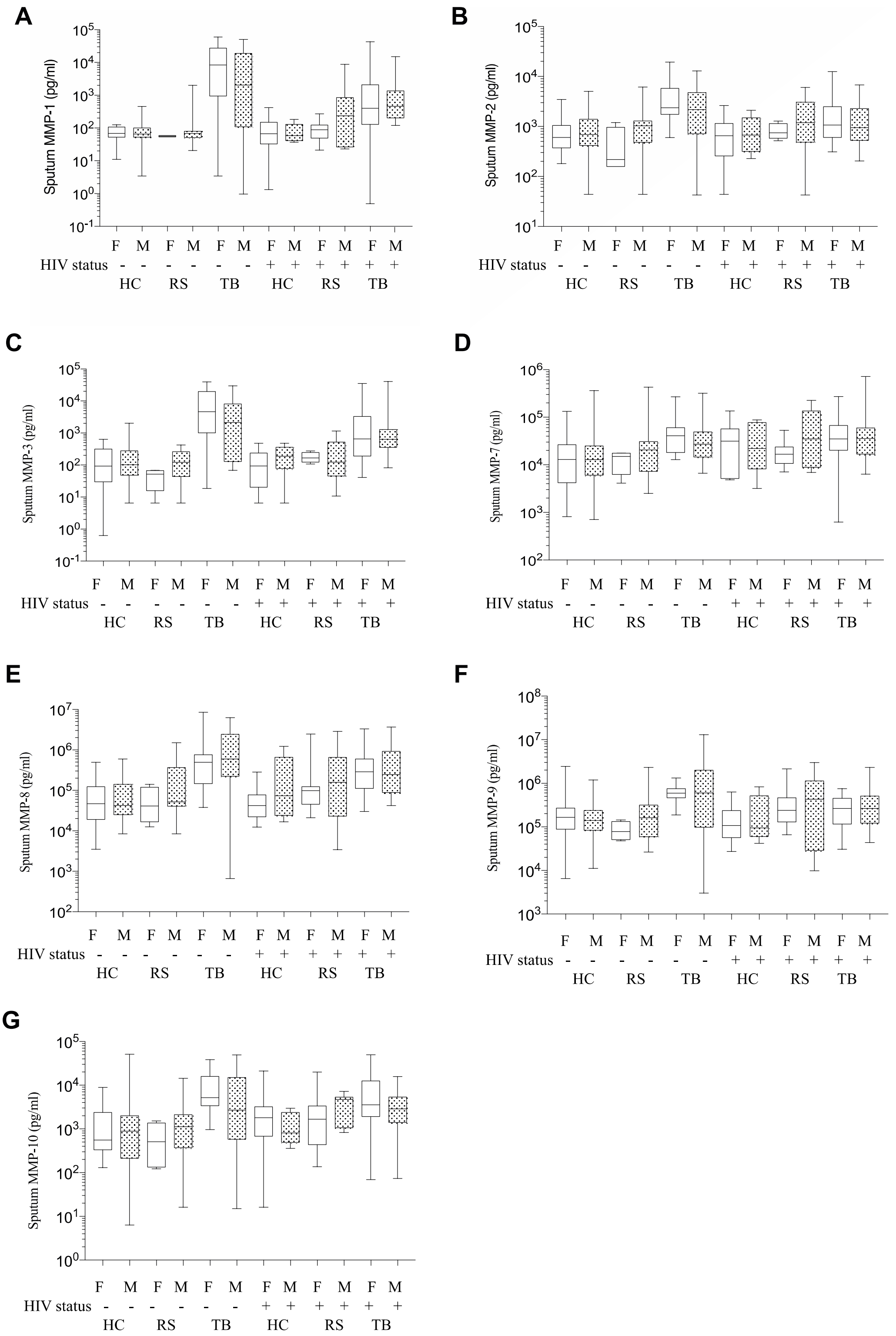

Supplementary Figure S2

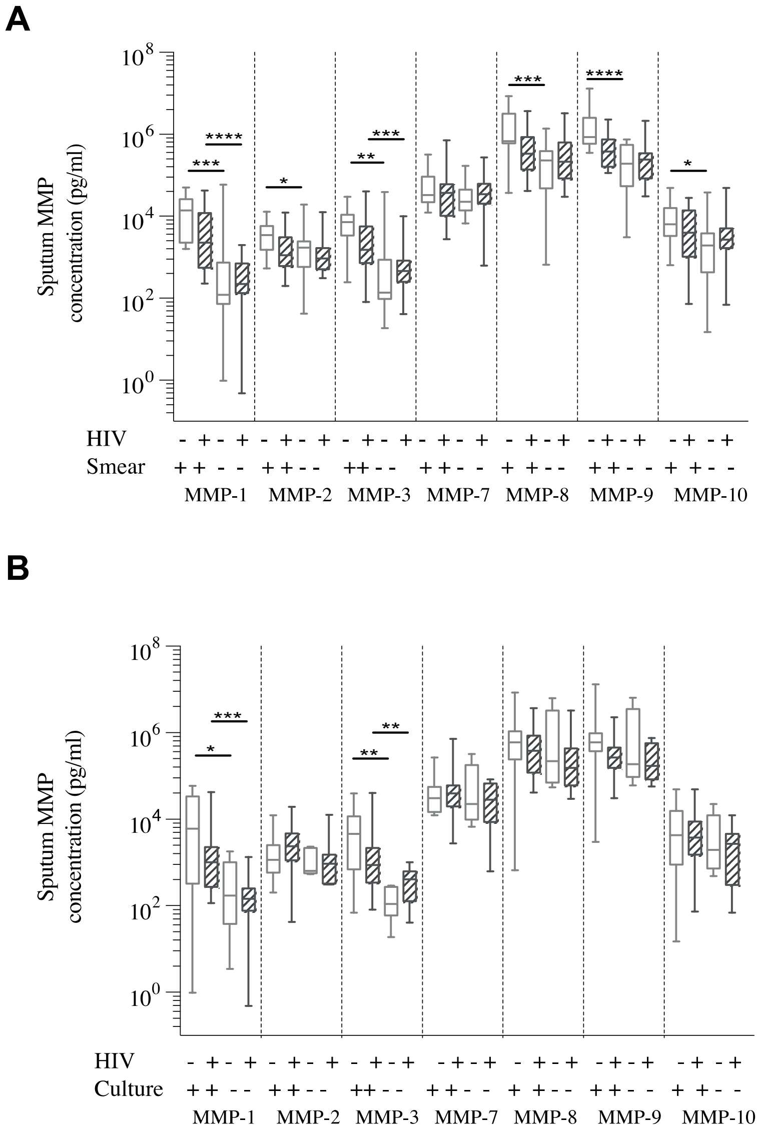

Supplementary Figure S3

A

r = 0.718 (0.583-0.814)
p <0.0001

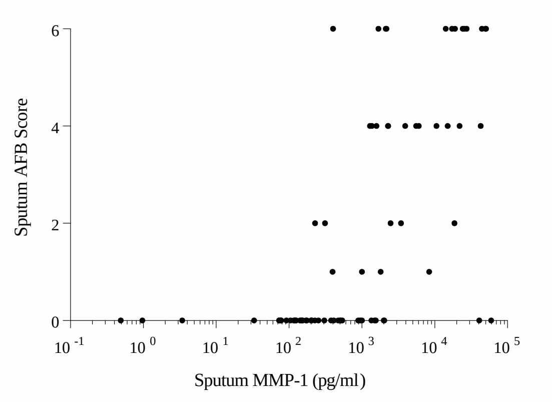

B

r = 0.631 (0.468-0.753)
p <0.0001

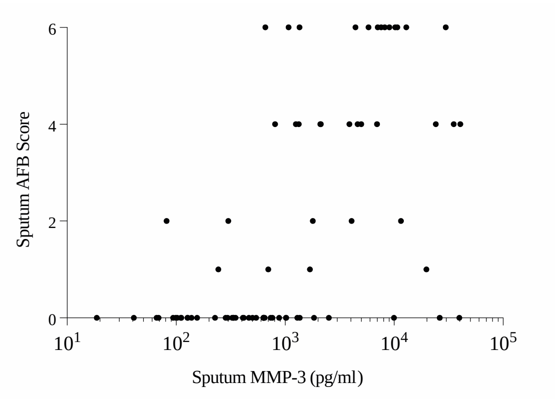

Supplementary Figure S4

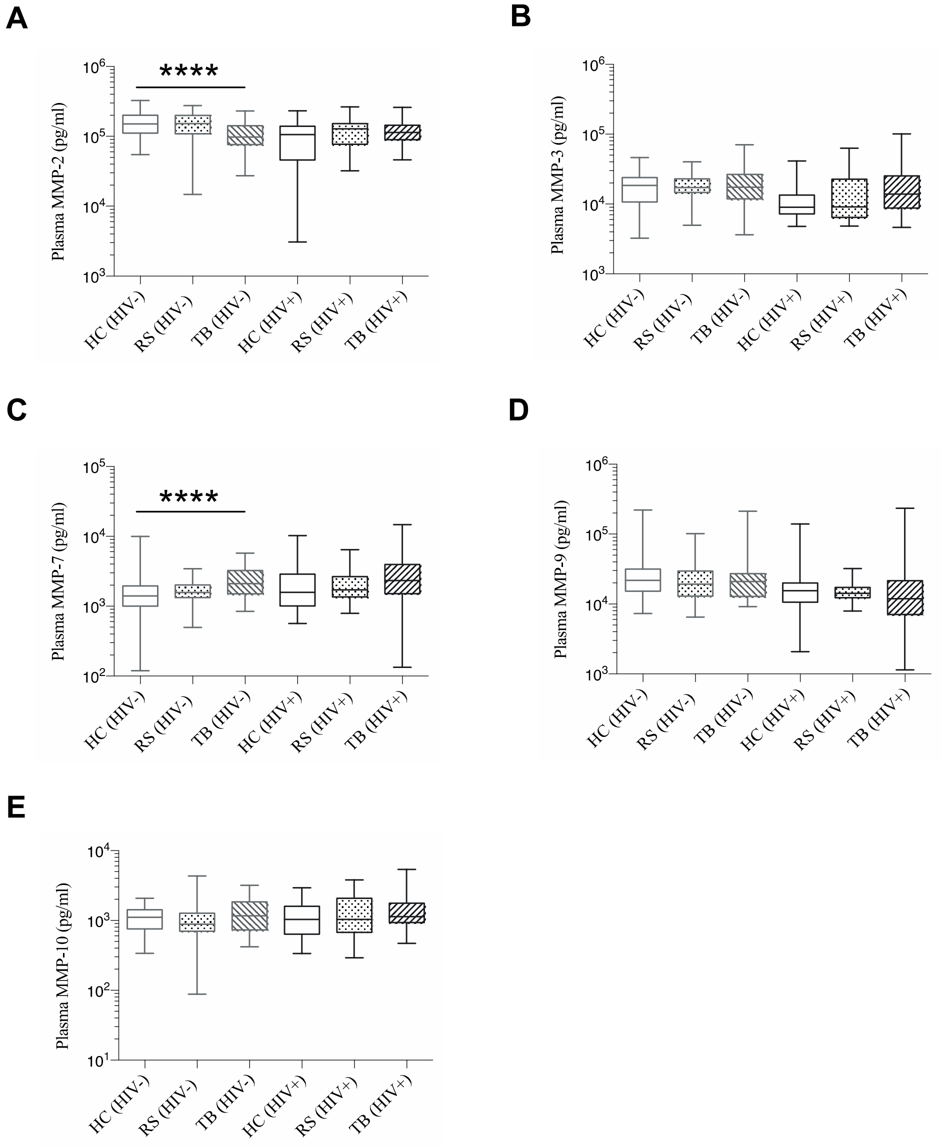

Supplementary Figure S5

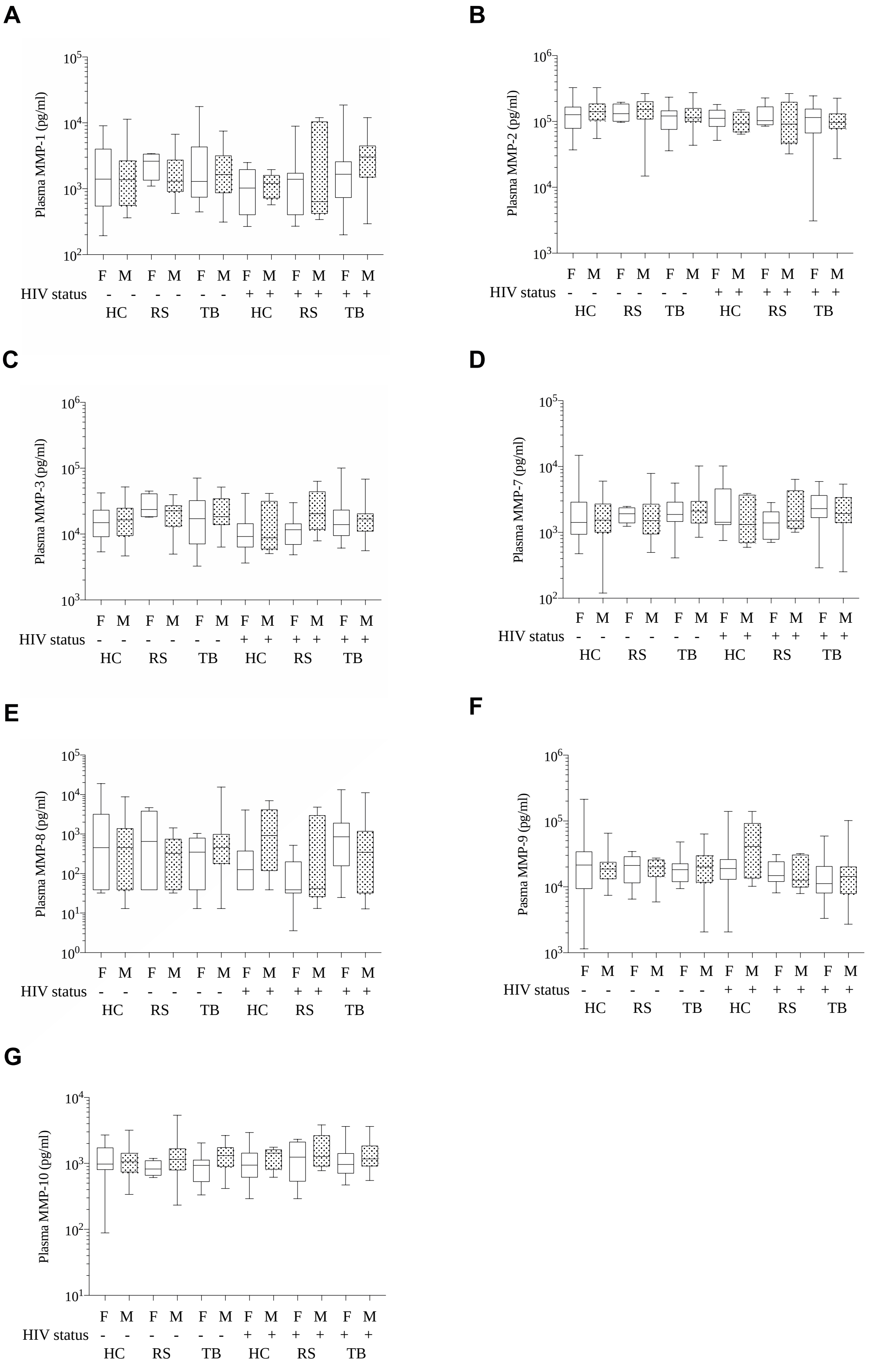

Supplementary Figure S6

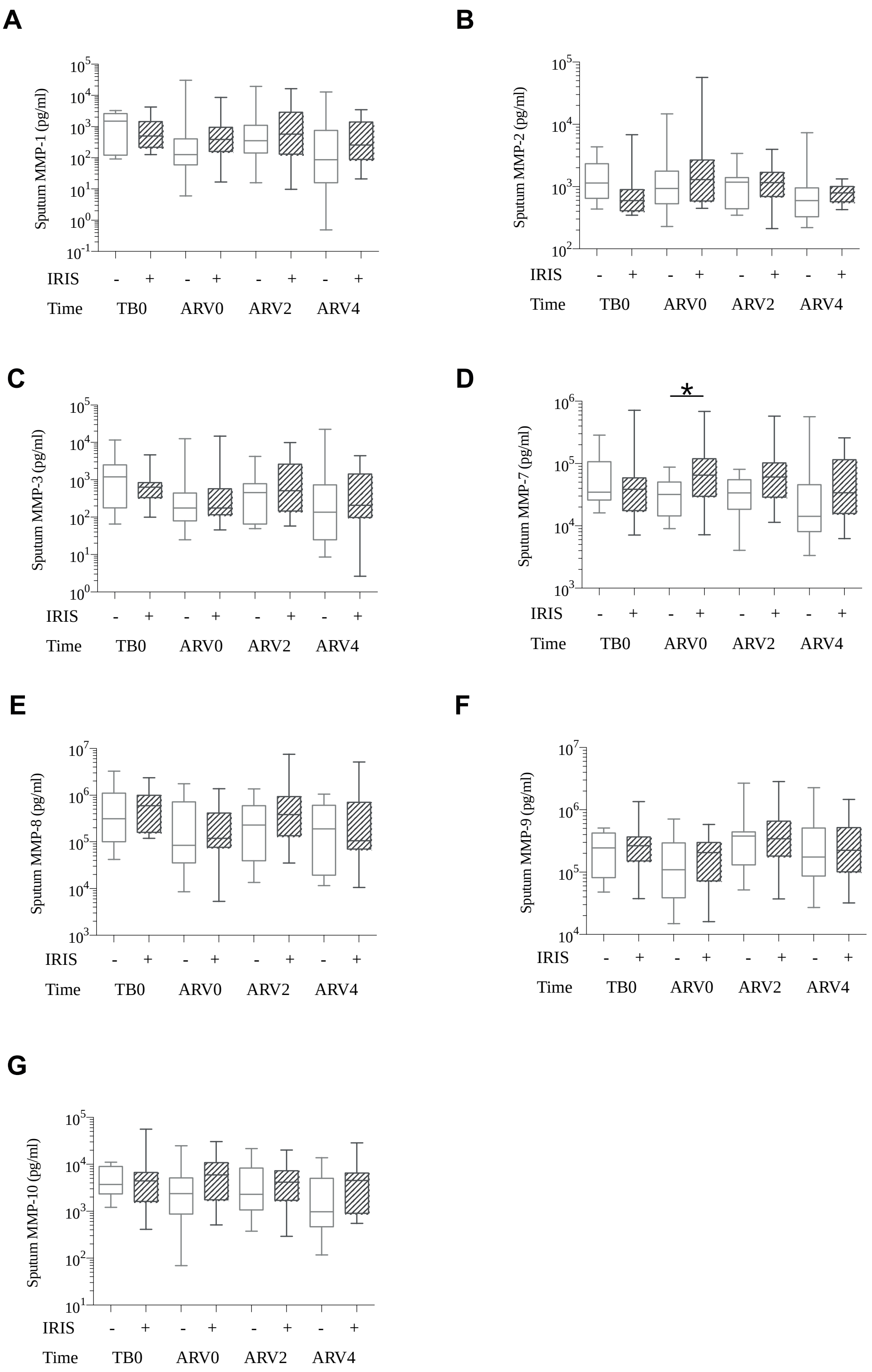

Supplementary Figure S7

Supplementary Tables

Supplementary Table S1 Diagnostic case definitions for Cross-sectional study

Designation	Description	Criteria	Respiratory Symptoms?	HIV status	Label
**TB patient**	TB	Sputum smear positive for AFB on microscopy OR<br><br>Gene Xpert positive for Mtb with suggestive clinical presentation OR<br><br>Sputum culture positive for Mtb on culture OR<br><br>Clinical features at enrolment highly suggestive of TB with diagnostic features on chest x-ray and decision taken to start patient on TB treatment by treating clinician*.	✓	+	TB (HIV+)
			✓	-	TB (HIV-)
**Control patient**	Respiratory symptomatic	All sputum samples obtained (minimum one induced sputum) smear negative for AFB AND<br><br>Gene Xpert negative for Mtb (if done) AND<br><br>All sputum cultures obtained (minimum one induced sputum) negative for Mtb AND<br><br>Low clinical suspicion for active TB after full clinical assessment, including review of chest radiographs where available AND<br><br>Not started on TB therapy	✓	+	RS (HIV+)
			✓	-	RS (HIV-)
	Healthy control	All sputum samples obtained smear negative for AFB AND<br><br>Gene Xpert negative for Mtb (if done) AND<br><br>All sputum cultures obtained negative for Mtb AND<br><br>Low clinical suspicion for active TB after full clinical assessment, including review of chest radiographs where available AND<br><br>Not started on TB therapy	X	+	HC (HIV+)
			X	-	HC (HIV-)

*following international guidelines[1,2]

Acid fast bacilli (AFB); *Mycobacterium tuberculosis* (Mtb); Tuberculosis (TB)

Supplementary Table S2 Microbiological diagnosis of TB.

HIV status	HIV-uninfected			HIV-infected			p value
Diagnostic category	Healthy control	Respiratory symptomatic	TB patient	Healthy control	Respiratory symptomatic	TB patient	TB (HIV-) vs TB (HIV+)
Frequency, n	61	24	39	16	17	53	N/A
Sputum smear positive for AFB, n (%)	0 (0)	0 (0)	22 (56)	0 (0)	0 (0)	17 (32)	0·016
Sputum culture positive for Mtb, n (%)	0 (0)	0 (0)	21 (54)	0 (0)	0 (0)	39 (72)	1·000
Median time to culture positivity, days (IQR)	N/A	N/A	10 (8-16)	N/A	N/A	17 (12-22)	0·004
Sputum Gene Xpert positive, n (%)	0 (0)	0 (0)	23 (59)	0 (0)	0 (0)	22 (42)	0·107
Sputum Gene Xpert negative, n (%)	0 (0)	1 (4)	1 (3)	1 (6)	ND	6 (11)	
Sputum not available, n (%)	6 (10)	0 (0)	3 (8)	0 (0)	0 (0)	2 (4)	0·647
No microbiological confirmation of TB diagnosis, n (%)	N/A	N/A	6 (15)	N/A	N/A	7 (13)	0·771

Supplementary Table S3 Unadjusted and adjusted linear regression models for the effect of HIV-1 infection and TB disease on log-transformed sputum MMP and plasma PIIINP concentrations. TB disease was associated with an increase in sputum MMP and plasma PIIINP concentrations in unadjusted and adjusted models, with the greatest difference (Diff.) for sputum MMP-1. TB disease in HIV-infected patients (HIV and TB) was associated with relatively lower sputum MMPs, but not with lower plasma PIIINP.

	Unadjusted									Adjusted*								
	**TB (no HIV)**			**HIV (no TB)**			**HIV and TB**			**TB (no HIV)**			**HIV (no TB)**			**HIV and TB**		
	Diff·	95% CI	p-value	Diff·	95% CI	p-value	Diff·	95% CI	p-value	Diff·	95% CI	p-value	Diff·	95% CI	p-value	Diff·	95% CI	p-value
**Sputum MMP-1**	3·26	2·55, 3·96	<0·001	0·17	-0·53, 0·87	0·624	-1·38	-2·43, -0·34	0·010	3·54	2·77, 4·32	<0·001	0·23	-0·52, 0·98	0·549	-1·70	-2·80, -0·59	0·003
**Sputum MMP-2**	1·16	0·71, 1·61	<0·001	0·10	-0·35, 0·55	0·674	-0·69	-1·36, -0·02	0·045	1·26	0·77, 1·76	<0·001	0·09	-0·38, 0·57	0·699	-0·81	-1·52, -0·11	0·024
**Sputum MMP-3**	3·08	2·40, 3·76	<0·001	0·32	-0·36, 1·00	0·351	-1·49	-2·16, -0·14	0·027	3·39	2·65, 4·12	<0·001	0·31	-0·40, 1·02	0·385	-1·51	-2·55, -0·46	0·005
**Sputum MMP-7**	0·94	0·47, 1·42	<0·001	0·49	0·01, 0·97	0·043	-0·61	-1·32, 0·10	0·093	0·87	0·33, 1·40	0·002	0·50	-0·02, 1·01	0·058	-0·64	-1·40, 0·11	0·096
**Sputum MMP-8**	1·98	1·39, 2·58	<0·001	0·34	-0·25, 0·94	0·258	-0·75	-1·64, 0·14	0·097	2·17	1·51, 2·83	<0·001	0·45	-0·18, 1·09	0·163	-1·01	-1·94, -0·07	0·035
**Sputum MMP-9**	1·27	0·78, 1·77	<0·001	0·20	-0·29, 0·70	0·419	-0·87	-1·61, -0·13	0·022	1·59	1·05, 2·13	<0·001	0·27	-0·25, 0·80	0·302	-1·19	-1·96, -0·41	0·003
**Sputum MMP-10**	1·58	0·93, 2·23	<0·001	0·79	0·13, 1·43	0·019	-0·97	-1·95, 0·00	0·050	1·59	0·89, 2·29	<0·001	0·65	-0·30, 1·32	0·061	-1·15	-2·15, -0·15	0·025
**Plasma PIIINP**	1·13	0·30, 1·95	0·008	0·50	-0·29, 1·28	0·210	0·32	-0·80, 1·43	0·572	1·09	0·23, 1·95	0·014	0·47	-0·33, 1·27	0·241	0·41	-0·74, 1·56	0·482

*adjusted for age, sex and smoking status

Supplementary Table S4 Differences in log MMP concentrations in females relative to males.

	Adjusted*		
	Diff.	95% CI	p-value
**Sputum MMP-1**	-0·14	-0·79, 0·52	0·680
**Sputum MMP-2**	-0·05	-0·46, 0·37	0·830
**Sputum MMP-3**	-0·25	-0·86, 0·37	0·078
**Sputum MMP-7**	-0·32	-0·77, 0·12	0·155
**Sputum MMP-8**	-0·44	-0·99, 0·12	0·121
**Sputum MMP-9**	-0·10	-0·55, 0·36	0·674
**Sputum MMP-10**	-0·29	-0·87, 0·30	0·340
**Plasma MMP-1**	-0·17	-0·54, 0·21	0·386
**Plasma MMP-2**	-0·05	-0·26, 0·17	0·673
**Plasma MMP-3**	-0·28	-0·52, -0·04	0·022
**Plasma MMP-7**	0·12	-0·16, 0·41	0·392
**Plasma MMP-8**	0·68	-0·05, 1·40	0·067
**Plasma MMP-9**	-0·06	-0·34, 0·22	0·667
**Plasma MMP-10**	-0·21	-0·43, 0·00	0·050

*adjusted for age, smoking status, HIV-1 infection and TB disease status

Supplementary Table S5 Association between induced sputum MMP concentrations and radiological findings assessed by Spearman rank-order correlation coefficient.

	All patients		TB (HIV-)		TB (HIV+)	
	r	p	r	p	r	p
**Cavity frequency**						
**MMP-1**	0·528	<0·0001	0·592	<0·0001	0·533	<0·0001
**MMP-2**	0·335	<0·0001	0·472	<0·0001	0·294	<0·001
**MMP-3**	0·502	<0·0001	0·565	<0·0001	0·484	<0·0001
**MMP-7**	0·109	0·146	0·151	0·090	-0·004	0·965
**MMP-8**	0·399	<0·0001	0·482	<0·0001	0·345	<0·0001
**MMP-9**	0·342	<0·0001	0·404	<0·0001	0·295	<0·001
**MMP-10**	0·188	0·012	0·212	0·017	0·075	0·383
**Chest x-ray inflammation score**						
**MMP-1**	0·601	<0·0001	0·452	0·011	0·220	0·212
**MMP-2**	0·381	<0·0001	0·335	0·065	0·294	0·091
**MMP-3**	0·553	<0·0001	0·453	0·011	0·253	0·149
**MMP-7**	0·350	<0·0001	0·078	0·678	0·187	0·289
**MMP-8**	0·554	<0·0001	0·308	0·092	0·337	0·051
**MMP-9**	0·408	<0·0001	0·306	0·094	0·186	0·292
**MMP-10**	0·327	<0·0001	0·262	0·154	-0·028	0·873

Supplementary Table S6 Demographics of the longitudinal cohort.

	Diagnosis		
	**non-IRIS**	**TB-IRIS**	**p value**
**Frequency, n**	18	29	N/A
**Median age, years (IQR)**	36 (31-43)	35 (30-42)	0·867
**Female, n (%)**	10 (56)	14 (48)	0·766
**Smoking history, current or ex-, n (%)**	4 (22)	9 (31)	0·739
**BCG vaccination, n (%)**	9 (50)	17 (59)	0·763
**Median days of TB treatment to ART initiation (IQR)**	22 (14-40)	15 (14-28)	0·127
**Required hospital admission, n (%)**	1 (6)	13 (45)	0·007

Anti-retroviral therapy (ART); interquartile range (IQR); not applicable (N/A); tuberculosis (TB); TB immune reconstitution inflammatory syndrome (IRIS).
P values are for Fisher's exact or Mann-Whitney U analysis.

Supplementary Table S7 Presenting clinical features of TB-IRIS in longitudinal cohort.

Presentation	n	%	Examples
**Pulmonary**	27	93	Shortness of breath, pleural effusion, worsening radiological features
**Constitutional**	29	100	Fever, night sweats, weight loss
**Gastrointestinal**	19	66	Abdominal pain, intra-abdominal lymphadenopathy, hepatomegaly
**Neurological**	4	14	Severe headache, seizure, meningitis
**Cardiac**	1	3	Myocarditis, pericardial effusion
**Nodal**	8	28	Tender, fluctuant lymphadenopathy
**Dermatological**	1	3	Rash
**Other**	1	3	Inflammatory arthritis

Supplementary Table S8 MMP concentrations in induced sputum and plasma in the longitudinal cohort

	Diagnostic category and sample timepoint							
	TB0		ARV0		ARV2		ARV4	
**Sputum**	**non-IRIS**	**TB-IRIS**	**non-IRIS**	**TB-IRIS**	**non-IRIS**	**TB-IRIS**	**non-IRIS**	**TB-IRIS**
Sample frequency n, (% enrolled participants)	8 (80)	13 (93)	14 (78)	23 (79)	11 (61)	17 (59)	14 (78)	13 (45)
**Median MMP-1, pg/ml (IQR)**	1491 (123-2625)	503 (218-1456)	127 (59-403)	391 (160-953)	354 (142-1118)	575 (130-2915)	87 (16-754)	255 (90-1409)
**Median MMP-2, pg/ml (IQR)**	1146 (650-2335)	597 (409-898)	936 (533-1764)	1296 (589-2683)	1181 (443-1396)	1163 (693-1690)	598 (330-954)	794 (573-1004)
**Median MMP-3, pg/ml (IQR)**	1199 (178-2529)	632 (332-830)	176 (80·5-445)	174 (116-582)	456 (66·1-791)	510 (146-2646)	137 (25-732)	208 (99-1437)
**Median MMP-7, pg/ml (IQR)**	34732 (26035-107564)	38808 (17487-58786)	31824 (14470-50602)	65463 (29760-119562)	33880 (18379-55301)	60731 (28786-103122)	14217 (8096-45956)	34192 (15912-115429)
**Median MMP-8, pg/ml (IQR)**	314589 (100810-1100000)	598292 (159413-999657)	84673 (35752-719541)	119014 (76539-415186)	229007 (39419-597879)	383157 (136111-929025)	190006 (19449-605951)	105939 (70850-701067)
**Median MMP-9, pg/ml (IQR)**	245854 (81844-425451)	264024 (152684-366811)	109865 (38895-294402)	205443 (72409-300945)	379195 (130253-443602)	343397 (182106-656466)	175029 (86195-512131)	223230 (101471-519814)
**Median MMP-10, pg/ml (IQR)**	3718 (2339-9056)	4421 (1621-6711)	2361 (872-5125)	5969 (1750-10882)	2283 (1062-8365)	4175 (1711-7259)	983 (464-5057)	4569 (903-6525)
**Plasma**								
Sample frequency, n (% enrolled participants)	10 (100)	14 (100)	17 (94)	29 (97)	17 (94)	25 (86)	16 (89)	18 (62)
**Median MMP-1, pg/ml (IQR)**	2971 (1806-3489)	4073 (2026-5148)	2123 (1152-3268)	3485 (1714-5721)	2194 (1099-5349)	4820 (2348-7115)	3166 (1446-4583)	3918 (2155-6524)
**Median MMP-2, pg/ml (IQR)**	151140 (134705-195738)	192297 (126289-243711)	219671 (156073-286765)	207052 (162984-291484)	211157 (148907-268753)	197745 (161620-265572)	209497 (179101-290903)	207084 (170303-282518)
**Median MMP-3, pg/ml (IQR)**	11146 (8003-15467)	23039 (14192-32693)	13689 (8455-17022)	17081 (11483-23055)	12025 (7373-19487)	17867 (12351-28800)	15846 (10129-19166)	17833 (12218-23420)
**Median MMP-7, pg/ml (IQR)**	3956 (3082-6329)	5327 (2359-9348)	3995 (2767-7853)	5163 (3719-7469)	4115 (3170-9818)	6802 (4989-11350)	4099 (2971-8055)	5420 (2938-11848)
**Median MMP-8, pg/ml (IQR)**	526 (326-2306)	4582 (2617-9090)	378 (353-1162)	946 (35-2617)	1095 (297-3929)	6104 (3702-12617)	1209 (137-5664)	3787 (1852-5862)
**Median MMP-9, pg/ml (IQR)**	8316 (4515-11231)	10051 (739-13214)	10203 (6601-16015)	8185 (6673-11185)	19182 (10673-35031)	17781 (10612-33208)	18168 (11974-36697)	15864 (11679-29911)
**Median MMP-10, pg/ml (IQR)**	1248 (1045-2821)	2153 (1770-2727)	1631 (1227-2530)	1967 (1386-2327)	1735 (1007-2374)	1801 (1247-2602)	1937 (1513-2745)	1487 (1224-2280)
**Median PIIINP, pg/ml (IQR)**	21651 (17757-33196)	43600 (30021-63913)	27333 (14057-33154)	25121 (18434-39468)	27093 (16616-41442)	39478 (26296-56996)	22424 (16892-31546)	46763 (29104-70641)

Interquartile range (IQR); matrix metalloproteinase (MMP); procollagen III N-terminal propeptide (PIIINP); tuberculosis immune reconstitution inflammatory syndrome (TB-IRIS).

Supplementary Table S9 Unadjusted and adjusted hierarchical linear regression models for the effect of IRIS on log-transformed sputum MMP concentrations.

	Unadjusted						Adjusted*					
	TB-IRIS			Timepoint			TB-IRIS			Timepoint		
	Diff.	95% CI	p-value	Diff.	95% CI	p-value	Diff.	95% CI	p-value	Diff.	95% CI	p-value
**Sputum MMP-1**	0·77	-0·31, 1·84	0·163	-0·23	-0·43, -0·02	0·033	0·76	-0·30, 1·82	0·161	-0·04	-0·43, -0·01	0·038
**Sputum MMP-2**	0·21	-0·22, 0·64	0·347	-0·14	-0·28, -0·00	0·048	0·24	-0·18, 0·66	0·259	-0·14	-0·28, -0·00	0·048
**Sputum MMP-3**	0·36	-0·54, 1·26	0·431	-0·24	-0·43, -0·05	0·012	0·36	-0·32, 1·26	0·428	-0·24	-0·43, -0·05	0·011
**Sputum MMP-7**	0·65	0·03, 1·27	0·040	-0·13	-0·27, 0·00	0·049	0·69	0·11, 1·27	0·019	-0·14	-0·27, -0·01	0·038
**Sputum MMP-8**	0·41	-0·37, 1·19	0·303	-0·16	-0·36, 0·04	0·111	0·38	-0·37, 1·13	0·321	-0·16	-0·36, 0·04	0·123
**Sputum MMP-9**	0·25	-0·31, 0·81	0·388	0·07	-0·08, 0·22	0·352	0·23	-0·33, 0·78	0·424	0·08	-0·07, 0·23	0·320
**Sputum MMP-10**	0·62	0·02, 1·22	0·044	-0·18	-0·36, -0·01	0·039	0·65	0·07, 1·23	0·029	-0·18	-0·35, -0·01	0·041

*adjusted for age, sex and smoking status

Confidence interval (CI); difference (Diff.); matrix metalloproteinase (MMP); tuberculosis immune reconstitution inflammatory syndrome (TB-IRIS).

Supplementary Table S10 Unadjusted and adjusted hierarchical linear regression models for the effect of TB-IRIS on log-transformed plasma MMP and PIIINP concentrations. TB-IRIS was associated with increased plasma MMPs, most significantly MMP-8, and increased plasma PIIINP in both adjusted and unadjusted models.

	Unadjusted						Adjusted*					
	TB-IRIS			Timepoint			TB-IRIS			Timepoint		
	Diff.	95% CI	p-value	Diff.	95% CI	p-value	Diff.	95% CI	p-value	Diff.	95% CI	p-value
**Plasma MMP-1**	0·38	0·01, 0·76	0·046	0·03	-0·06, 0·12	0·491	0·34	-0·01, 0·69	0·054	0·03	-0·05, 0·12	0·457
**Plasma MMP-2**	-0·06	-0·30, 0·19	0·645	0·02	-0·01, 0·04	0·239	-0·05	-0·29, 0·19	0·691	0·02	-0·01, 0·04	0·235
**Plasma MMP-3**	0·37	0·07, 0·67	0·015	0·00	-0·05, 0·05	0·966	0·35	0·08, 0·62	0·012	-0·00	-0·05, 0·05	0·960
**Plasma MMP-7**	0·22	-0·16, 0·59	0·263	0·12	0·07, 0·16	<0·001	0·23	-0·15, 0·60	0·240	0·12	0·07, 0·16	<0·001
**Plasma MMP-8**	1·26	0·40, 2·12	0·004	0·41	0·14, 0·68	0·003	1·27	0·45, 2·10	0·002	0·42	0·15, 0·69	0·002
**Plasma MMP-9**	0·01	-0·30, 0·31	0·958	0·33	0·23, 0·43	<0·001	0·00	-0·30, 0·30	0·991	0·33	0·23, 0·43	<0·001
**Plasma MMP-10**	0·11	-0·12, 0·34	0·336	-0·03	-0·06, 0·01	0·132	0·1	-0·13, 0·33	0·385	-0·03	-0·06, 0·01	0·128
**Plasma PIIINP**	0·46	0·19, 0·72	0·001	0·09	0·00, 0·17	0·041	0·47	0·21, 0·73	<0·001	0·09	0·00, 0·17	0·047

*adjusted for age, sex and smoking status

Confidence interval (CI); difference (Diff.); matrix metalloproteinase (MMP); procollagen III N-terminal propeptide (PIIINP); tuberculosis immune reconstitution inflammatory syndrome (TB-IRIS).

Supplementary Table S11 Unadjusted and adjusted logistic regression models for the effect of TB-IRIS and study timepoint on odds of LAM detection in urine. An increased odds of LAM detection in urine was found for TB-IRIS compared to non-IRIS patients and for later compared to earlier study timepoints.

	Unadjusted						Adjusted*					
	**TB-IRIS**			**Timepoint**			**TB-IRIS**			**Timepoint**		
	OR	95% CI	p-value	OR	95% CI	p-value	OR	95% CI	p-value	OR	95% CI	p-value
**Urine LAM positive**	10·59	0·99, 113·49	0·051	0·60	0·37, 0·98	0·039	10·87	1·02, 115·88	0·048	0·60	0·37, 0·97	0·039

*adjusted for age, sex and smoking status

Confidence interval (CI); lipoarabinomannan (LAM); odds ratio (OR); matrix metalloproteinase (MMP); procollagen III N-terminal propeptide (PIIINP); tuberculosis immune reconstitution inflammatory syndrome (TB-IRIS).
